# Adaptation of a Mice Doppler Echocardiography Platform to Measure Cardiac Flow Velocities for Embryonic Chicken and Adult Zebrafish

**DOI:** 10.3389/fbioe.2019.00096

**Published:** 2019-05-14

**Authors:** Fatiha M. Benslimane, Maha Alser, Zain Z. Zakaria, Anju Sharma, Hana A. Abdelrahman, Huseyin C. Yalcin

**Affiliations:** ^1^Biomedical Research Center, Qatar University, Doha, Qatar; ^2^Department of Biological and Environmental Sciences, College of Arts and Science, Qatar University, Doha, Qatar

**Keywords:** Doppler echocardiography, blood flow velocity, chick embryo, zebrafish, cardiac function, mechanobiology

## Abstract

Ultrasonography is the most widely used imaging technique in cardiovascular medicine. In this technique, a piezoelectric crystal produces, sends, and receives high frequency ultrasound waves to the body to create an image of internal organs. It enables practical real time visualization in a non-invasive manner, making the modality especially useful to image dynamic cardiac structures. In the last few decades, echocardiography has been applied to *in vivo* cardiac disease models, mainly to rodents. While clinical echocardiography platforms can be used for relatively large animals such as pigs and rats, specialized systems are needed for smaller species. Theoretically, as the size of the imaged sample decreases, the frequency of the ultrasound transducer needed to image the sample increases. There are multiple modes of echocardiography imaging. In Doppler mode, erythrocytes blood flow velocities are measured from the frequency shift of the sent ultrasound waves compared to received echoes. Recorded data are then used to calculate cardiac function parameters such as cardiac output, as well as the hemodynamic shear stress levels in the heart and blood vessels. The multi-mode (i.e., b-mode, m-mode, Pulsed Doppler, Tissue Doppler, etc.) small animal ultrasound systems in the market can be used for most *in vivo* cardiac disease models including mice, embryonic chick and zebrafish. These systems are also associated with significant costs. Alternatively, there are more economical single-mode echocardiography platforms. However, these are originally built for mice studies and they need to be tested and evaluated for smaller experimental models. We recently adapted a mice Doppler echocardiography system to measure cardiac flow velocities for adult zebrafish and embryonic chicken. We successfully assessed cardiac function and hemodynamic shear stress for normal as well as for diseased embryonic chicken and zebrafish. In this paper, we will present our detailed protocols for Doppler flow measurements and further cardiac function analysis on these models using the setup. The protocols will involve detailed steps for animal stabilization, probe orientation for specific measurements, data acquisition, and data analysis. We believe this information will help cardiac researchers to establish similar echocardiography platforms in their labs in a practical and economical manner.

## Introduction

Cardiovascular diseases (CVDs) are disorders of the heart and blood vessels. They are the leading cause of mortality, constituting 31% of all fatalities globally (Kendir et al., 2018). CVDs can occur prenatally, known as congenital heart defects (CHDs), or develop at later stages of life. CHDs account for around 25% of all human congenital abnormalities (Roger et al., 2011) and it affects 1–2% of infants globally. Although prenatal cardiac malformations are linked to genetics, the etiology is highly complex and involves multiple factors (Lindsey et al., 2014). Recently, it has been shown that genetics accounts for < 20% of heart defects. Other environmental factors, such as hyperglycemia during maternal diabetes, or disturbed hemodynamics are thought to play a crucial role in CHD development (Midgett et al., 2017). Hemodynamics are the mechanical forces applied by blood flow, such as pressure or shear stress. Primarily, blood is pumped during development and remodeling, which suggests that hemodynamics governs cardiac development (Hove et al., 2003; Forouhar et al., 2006; Culver and Dickinson, 2010; Yalcin et al., 2011). The constant interactions between blood flow dynamics and cardiac tissue motion signals the endothelial cells lining the chambers and the valves. Deviations from normal hemodynamic conditions lead to cardiac malformations as the heart is very sensitive to biomechanical cues at the early embryonic stages (Goenezen et al., 2012). Clinical observations and animal experiments have shown that when hemodynamics are disturbed, fetal cardiac defects develop, consequently leading to CHDs at birth (Goenezen et al., 2012). Disturbed hemodynamics also contribute to formation of CVDs that develop later in life. For instance, it was shown that heart valve/blood vessel calcification localize to flow regions (i.e., oscillatory flow regions) that deviate from normal hemodynamics (Balachandran et al., 2011; Mahler et al., 2014; Fernández Esmerats et al., 2016; Amindari et al., 2017).

Animal models are very useful to investigate how disturbed hemodynamics contribute to CVDs (Zaragoza et al., 2011). Their use in relevant research facilitated to unravel various aspects of the diseases including etiology, pathophysiology, progression, and underlying biological pathways. Consequently, this knowledge led to the advancement of new diagnostic techniques and the discovery of new potential therapeutic approaches (Chorro et al., 2009). Vertebrate species, particularly, are favored models because of their highly conserved developmental processes. Their lifespan is relatively short, by 3 months they are considered as adults and in captivity they reach to 2 years of age, which allows investigators to monitor the disease at an accelerated pace. The genetically modified models that can be developed allows the rapid establishment of proof-of-principle (Camacho et al., 2016). For instance, rodent knockouts models have been extensively used for assessing the effects of genes on normal cardiac development and CVDs (Phoon et al., 2004; Bruneau, 2008). Furthermore, rodent models are used to assess the mother's nutrition effects on cardiovascular conditions and placental development on embryonic growth as well as cardiac formation (James et al., 1998; Yu et al., 2008). However, rodent embryos are not considered ideal models to study hemodynamic effects on cardiac development; they lack the ability to develop *ex utero* beyond early stages and accessing the embryo *in utero* during development is challenging (Piliszek et al., 2011). Additionally, certain genetic knockdowns are lethal either during embryogenesis or at the early stage of adulthood limiting the ability of the investigator to understand its molecular mechanism, which in turn limits the window for developing therapeutics. It is for all of these reasons that zebrafish and embryonic avian models have been more widely used to monitor hemodynamic conditions throughout development. In the case of adult zebrafish, these investigations extend to include some genes that cause severe phenotypes or are lethal in mammals (Hove et al., 2003; Jenkins et al., 2010; Lindsey et al., 2014; Yalcin et al., 2017; Yalcin, 2018). Although these models are being used extensively by researchers, the available cardiac imaging systems that allow the study of cardiac function in small animals are complex and highly expensive.

Doppler echocardiography is a popular tool for assessing cardiac function. The technique enables the measurement of blood velocities through blood vessels, heart valves, and cardiac chambers, which is then used to diagnose CVDs (Spencer et al., 2013). Doppler echocardiography is also very useful to apply to *in vivo* CVD models to investigate disturbed hemodynamics. Most current small imaging echocardiography platforms are designed for mouse studies and can be applied to other animal models. For example, previously, we have adapted B-mode-guided Doppler ultrasound visual sonic *in vivo* 770 platform (Vevo 770, Visualsonics, Inc., Toronto, Candad) for embryonic chick studies and documented evolving atrio-ventricular canal and outflow tract (Yalcin et al., 2011; Bharadwaj et al., 2012). Such high cost systems involve multiple modalities including Doppler, m-mode, b-mode, and tissue strain. Alternatively, there are also lower cost single mode Doppler echocardiography platforms, used mainly for mice imaging. Adaptation of these mice Doppler systems for use in other animal models requires testing and evaluation of these platforms. Here, we explain how we have adapted a mice Doppler echocardiography system to embryonic chick and adult zebrafish studies. We believe this information will help cardiac researchers to establish similar echocardiography platforms in their labs in a practical and economical manner.

The studies were carried out in accordance with the recommendations of “Use of Zebrafish” and “Use of Avian Embryos” policies, Qatar University—Institutional Animal Care and Use Committee (QU-IACUC). The protocol was approved by the QU-IACUC.

## Theory of Blood Velocity Measurements via Doppler Echocardiography

Typically, echocardiography imaging systems have three modes: b-mode, m-mode, and Doppler. B-mode (brightness-mode) and M-mode (motion-mode) are used for the assessment of morphology and movement of the tissue, whereas Doppler mode is used for the evaluation of cardiac function (Gregg and Butcher, 2012). More specifically, Doppler mode is used to measure blood flow velocity and determine flow direction. The technique is based on detecting the change in the frequency of sound waves that occur as they are reflected off a moving object, known as Doppler shift. Doppler echocardiography *in vivo* measures blood flow by detecting the frequency shift due to movement of erythrocytes (Gregg and Butcher, 2012; Kowalski et al., 2014). In this technique, a piezoelectric crystal produces and sends short impulse high frequency ultrasound waves to the body. Blood flow velocities are then calculated from the sound waves as scattered echoes received by the same crystal. It enables practical real time flow measurement in a non-invasive manner, making the modality especially useful to image dynamic cardiac flows. The frequency required reflects the size of the sample, as the size of the sample to be imaged decreases, the frequency of the ultrasound transducer increases. The clinical platforms available have transducers with maximum frequency of about 15 MHz, while advanced imaging systems can go up to 90 MHz making these systems more expensive.

**Figure 1:**
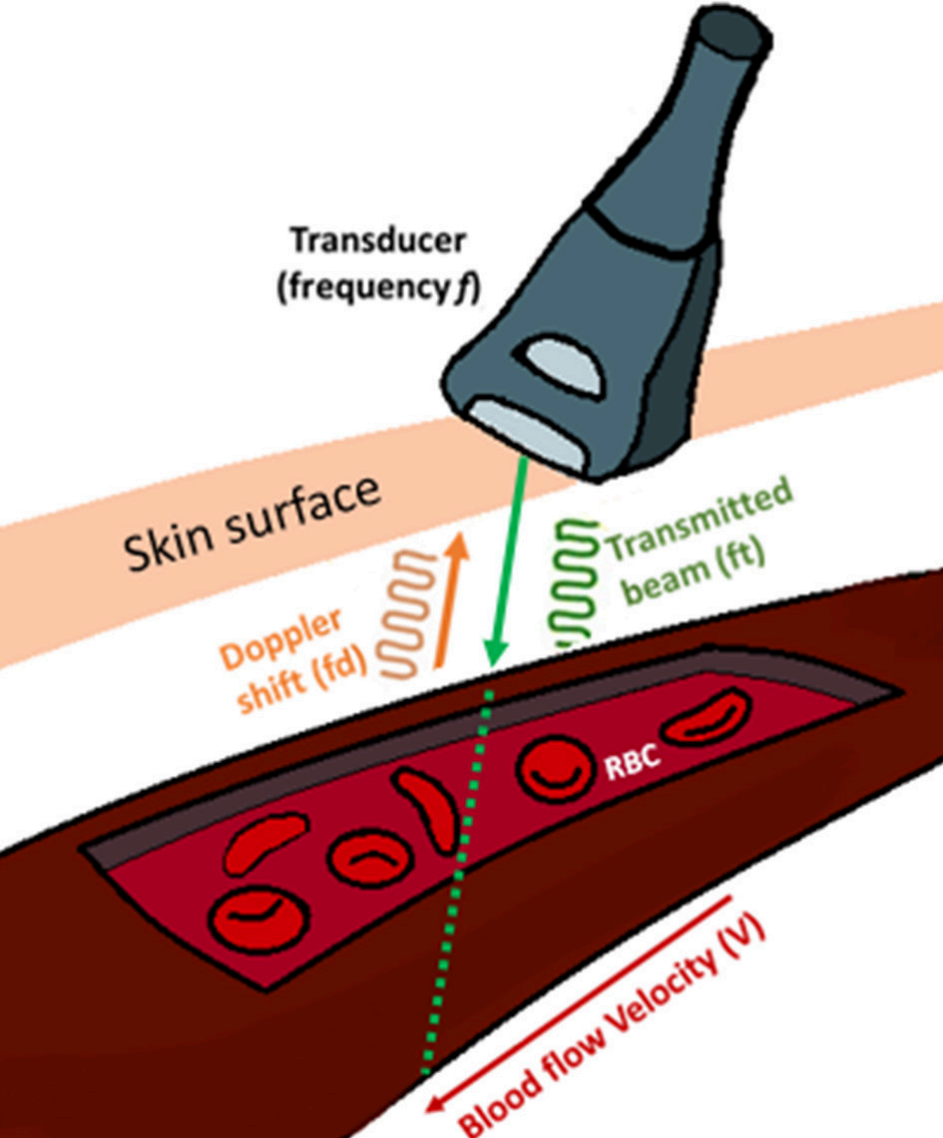
Blood flow velocity (V) measurement using an ultrasound transducer. Doppler signal transmits a frequency (F_t_) and receives the backscattered signals from the moving erythrocytes within the vessel at a frequency (F_d_) known as Doppler shift. The angle (θ) is the angle between the transmitted signal and blood flow.

Doppler mode utilizes real time spectral display, also known as waveforms. These spectral waveforms are indication of the dynamic nature of the blood flow through the heart and they reflect the elasticity of heart chambers and blood vessels. Therefore, obtaining these waveforms are very useful to monitor the cardiac function. The spectra is generated based on the Doppler shift according to Equation (1), where *fd* is the Doppler shift, *ft* is the transmitted beam, *V* is the velocity of the blood, θ is the angle between the transducer and the blood flow direction and *c* is the speed of sound in tissue (Figure 1).

(1) $$\begin{array}{r} Doppler\;Frequency\;\left(fd\right)=\;\frac{2\;.\;ft\;.\;V\;.\;\cos\theta }{c} \end{array}$$

**Figure 2:**
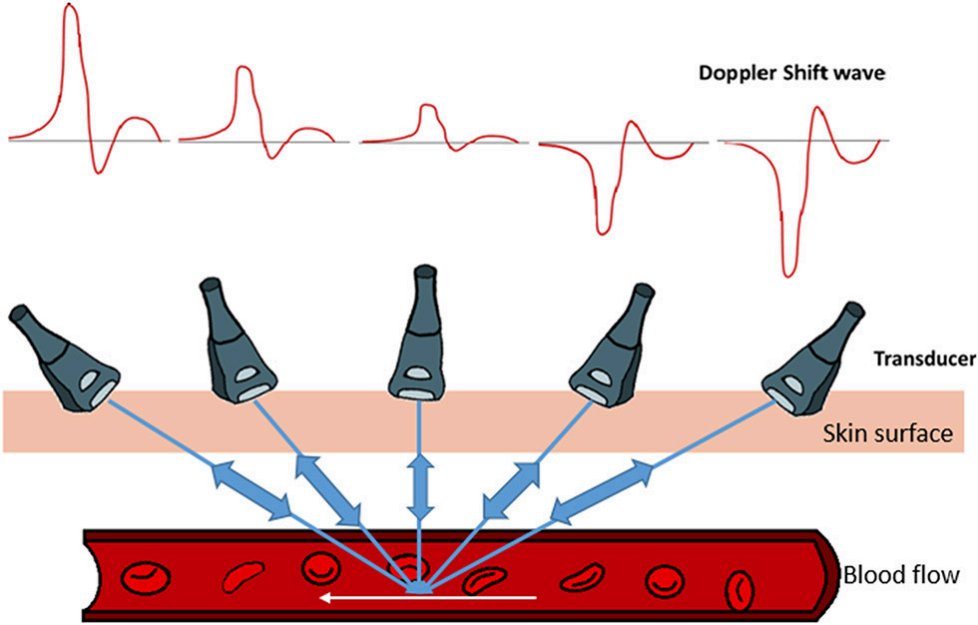
Graphical demonstration of the relationship between Doppler's shifted frequency with respect to the insonation angle of Doppler's ultrasound beam. Pointing the Doppler ultrasound beam toward the direction of vessel's blood flow results in observing a positive Doppler shifted signal. As the Doppler ultrasound beam points away from the blood flow direction, a negative Doppler shift signal is noted. The smaller the angle between the blood vessel flow and Doppler ultrasound beam, the larger the Doppler shift signal. As the Doppler ultrasound beam approaches a 90° angle, very small signals are produced. Therefore, for any given blood flow, the larger the angle, the smaller the Doppler shift.

Here, the factor two is for the round trip of the traveling sound waves, from the transducer, hitting the sample and reflecting back to the transducer. The direction of blood flow and the Doppler wave angle between the ultrasound beam and vessel are important factors in determining the velocity (Figure 2). The bigger the angle the more interference with the recorded spectra. To obtain the largest Doppler shift using ultrasound, the transducer must be placed at an angle of zero degrees to the vessel of interest, however due to practical considerations, clinically the angle is placed at around 60 degrees. For animal experimentation, because of the small sizes of the animals, it is easier to align the probe with flow so that the angle is zero (please see relevant sections below and Figure 2). The direction of the blood flow, away or toward, the transducer is another important factor that interferes with the way the signal is digitized (Figure 2). Once the spectrogram is obtained, the blood flow velocities can be extracted from the waveforms and the heart rate is calculated by the frequency analysis of velocity waveform (Kowalski et al., 2014).

Doppler imaging modality is practical, and inexpensive in comparison with other modalities like Magnetic resonance imaging (MRI), Computed tomography (CT) and Optical coherence tomography (OCT). In addition, Doppler platforms are portable, facilitating image acquisition. Echocardiography is completely non-invasive and it has not been associated with any adverse effects (Spencer et al., 2013). Furthermore, Doppler blood velocity parameters are particularly important in direct translational studies. Cardiac parameters that are gathered with other techniques include, the heart weight, left ventricular volume, stroke volume, cardiac output, and aortic diameter are all proportional to body weight. This means that as the body size changes these parameters vary. As such, translating some of these measurements to other larger species or even humans would be difficult and direct translation would be challenging. On the other hand, aortic velocity and pulse wave velocity that can be obtained via Doppler are independent of body size. Aortic velocity and pulse wave velocity of a mouse, rat or a human, without the timing scale, are very similar. Their values across species do not vary that much allowing for a direct translation (Dawson, 1991).

Doppler echocardiography has been widely used in relatively larger mammalian animal models for *in vivo* cardiac function assessment (Watson et al., 2004; Locatelli et al., 2011). However, studying a small organism with a length size that ranges from 20 to 40 mm is challenging, nonetheless, it is now possible through the use of advanced high-frequency ultrasonography (up to 70 MHz, 30 μm axial resolution). The use of high frequency echocardiography in assessing cardiac function in small animals has recently begun to be explored. However, standardized approaches for image acquisition and data analysis are critically lacking. To date, reported studies displayed substantial differences in the methodologies including the choice and concentration of anesthetic agent, scanning environment and scanning views and analyzing techniques. Furthermore, there was limited data on reproducibility and quality control (Ho et al., 2002; Sun et al., 2008; Parente et al., 2013; González-Rosa et al., 2014; Lee et al., 2014; Hein et al., 2015; Huang et al., 2015; Kang et al., 2015; Wilson et al., 2015; Ernens et al., 2016). There are several systems available that could be utilized for Doppler echocardiography analysis for small animal imaging. In this study, we aimed to adapt a commonly used mice Doppler platform for embryonic chick and adult zebrafish models, which are two common models of cardiac research. Below are the details of our image acquisition and image analysis practice using the system.

### Mice Doppler System

**Figure 3:**
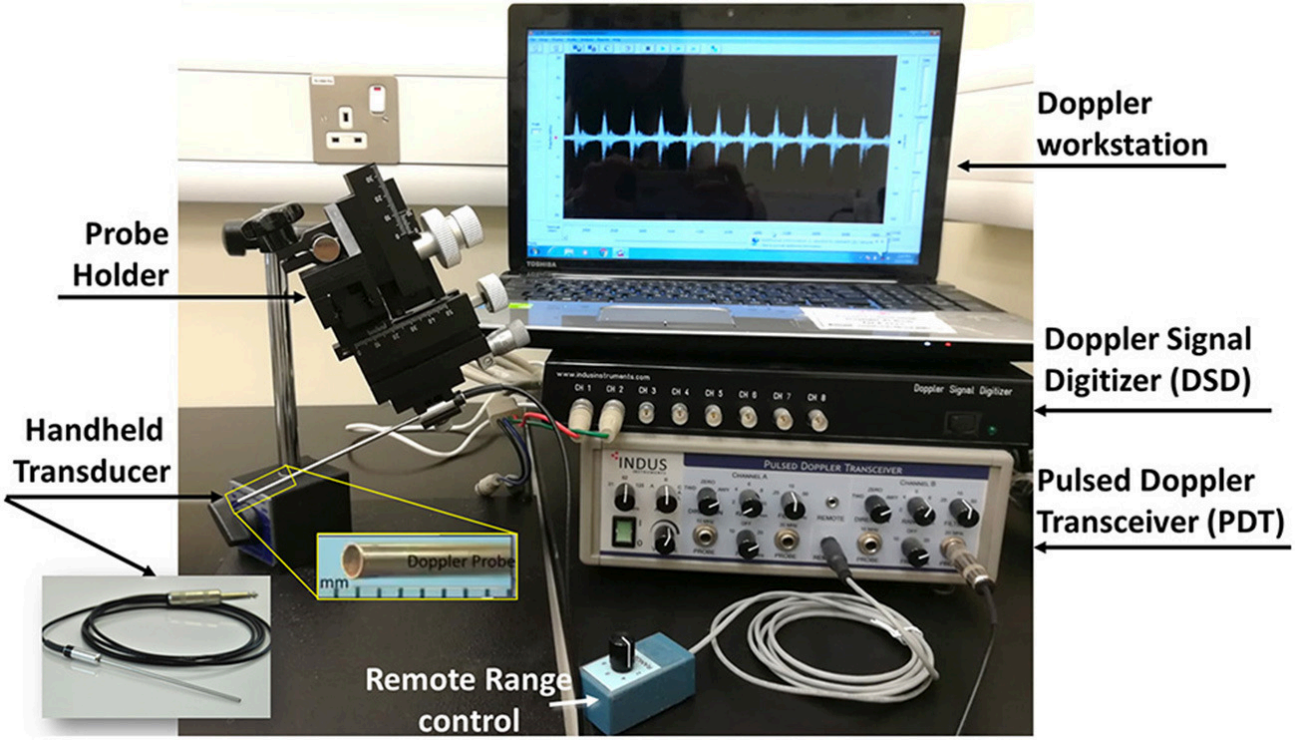
Mice Doppler system. The system is composed of a Doppler work station, signal digitizer, transceiver, a remote range control, and a handheld transducer. The interface shows a spectrogram of a signal containing a frequency of 20 kHz.

There are multiple Doppler systems used primarily on anesthetized mice and rats for noninvasive evaluation of the cardiovascular physiology (Hinton et al., 2016). They allow investigators to follow changes that occur due to disease progression, remodeling, aging, and the effects of pharmacological or surgical interventions. These systems consists of four components: two hardware component boxes, the Pulsed Doppler Transceiver (PDT) and Doppler Signal Digitizer (DSD), as well as a Handheld transducer and Doppler Workstation (DW). Here, as an example, we will present Indus Doppler System (Indus Instruments, USA.). System components and data acquisition for other similar systems do not differ. The transceiver has two channels and the digitizer takes the signal and transfers it to the computer. The PDT channels gives the option to set the direction of the flow, the range, the filters and the type of the transducer probe used. The range is the distance from the tip of the transducer probe to the location where velocity is measured and filter is selected to minimize noise in the measurement. There are three different probes available for measurements: 5, 10, and 20 megahertz (MHz). We have added a probe holder for micromanipulations (WPI 3301 micromanipulator), to facilitate the probe's orientation, and stabilization. The system component is displayed in Figure 3. As mentioned above, the angle is quite important when calculating the velocity. Ideally, it is preferred to fade to zero so that it has no effect on the velocity calculation. This means the Doppler probe has to be aligned with the blood flow as much as possible either with the blood moving toward or away from the probe. The probe on the Indus system is quite small, 1.0 mm diameter, which facilitates aligning it with the flow without creating an angle (Figure 3). The software setup and the data analysis are similar however, Doppler velocity measurements, data acquisition using this system will differ according to the species studied. The reason is that the structure and location of the heart and blood vessels differ across the species. The details of the measurements for the chick embryo and zebrafish will be discussed later on in this paper.

**Figure 4:**
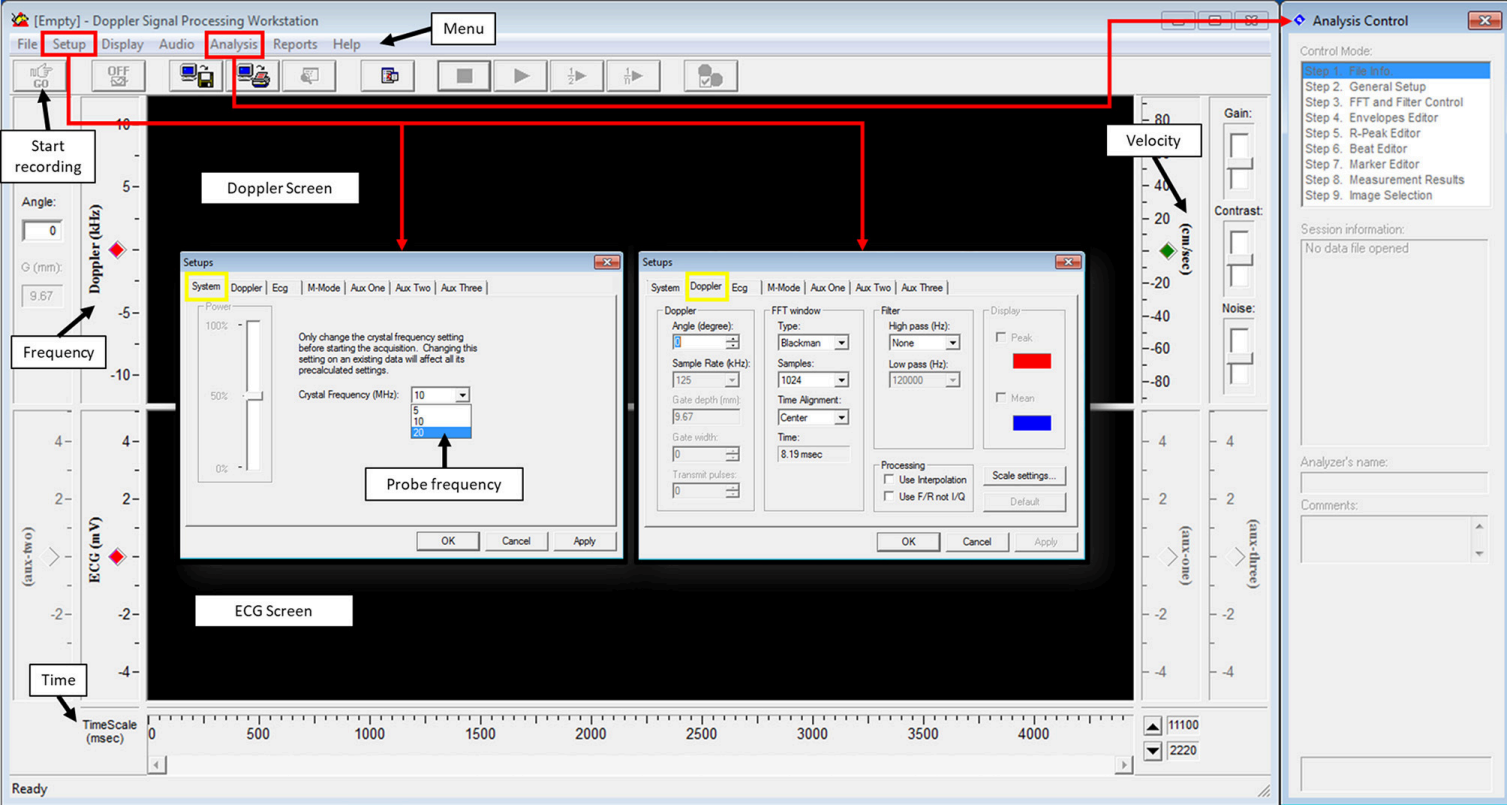
Doppler signal processing workstation user's interphase.

Doppler Signal Processing Workstation software is used to process the signal from the DSD. One important parameter that needs to be set prior to acquisition is the crystal frequency; this will depend on the probe being used (Figure 4, setups, system). Another important parameter that needs to be adjusted, although not important during acquisition but crucial during analysis, is the angle between the probe and the blood flow direction (Figure 4, Setups, Doppler, angle). Fast Fourier transform (FFT) parameters in Doppler setting tab controls the way the signal is presented (Figure 4, Setups, Doppler, FFT window). For best image signal, specifically for applications presented here, Blackman view along with central alignment and 1,024 samples can be selected. No high pass filter is needed but a low pass filter of 120,000 Hz is appropriate. These settings can be found from the setup menu under system and Doppler tabs. Finally, the length of the recorded signal has to be identified. This can be done from the setup menu under options. Once these parameters are set, acquisition can be started to obtain a spectrogram.

**Figure 5:**
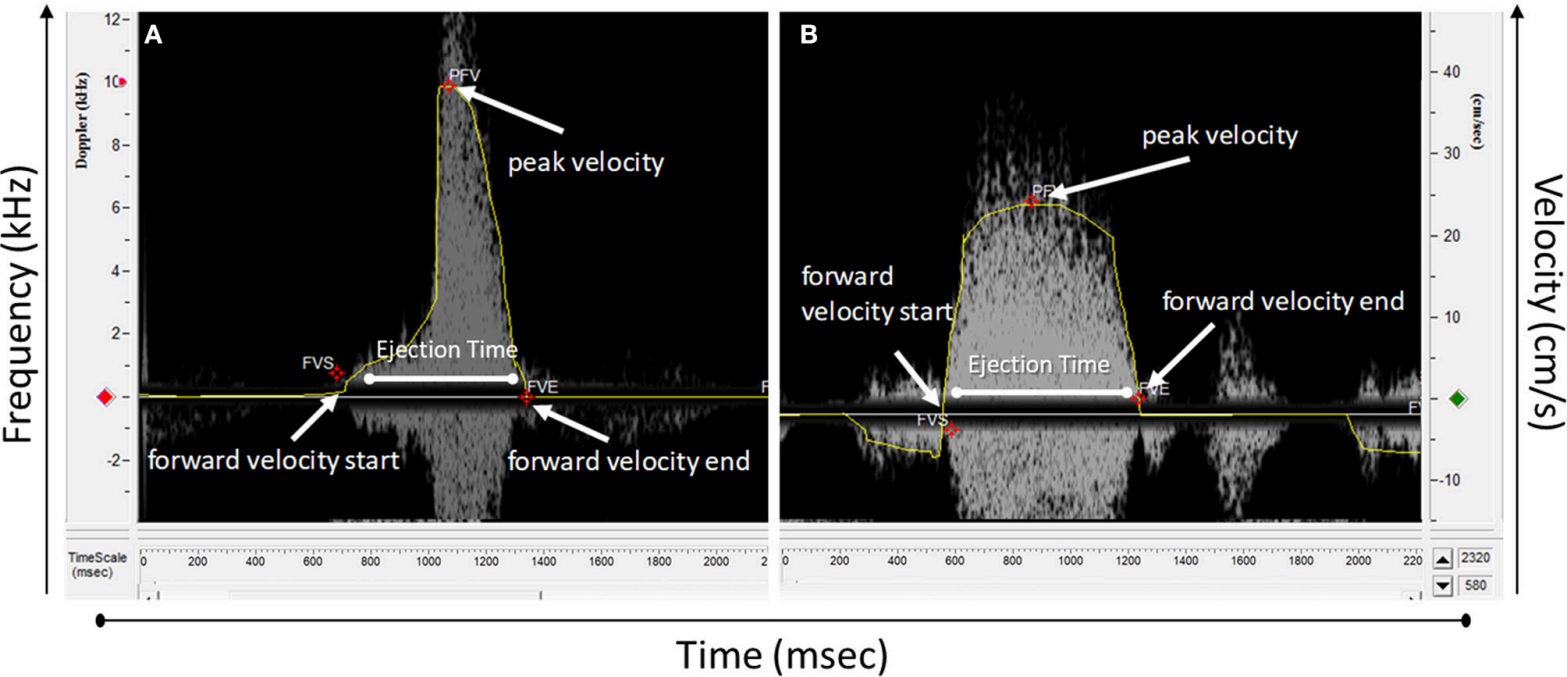
Doppler spectrograph waveform analyzed using Doppler workstation software. **(A)** A wave from an atrioventricular valve and **(B)** outflow track valve.

The spectrogram obtained can then be analyzed using an analysis software. Usually, software provided with the system for data acquisition is also used for data analysis. For the Indus software, from the analysis menu, analysis control window is opened to process the waveforms (Figure 4). The software can automatically detect the envelopes, tracing the edges of the Doppler spectrogram (Figure 5, yellow line), which will then be exported as a data file to be further processed. Here we will present how to make measurements for blood flow through the heart valves. Some important parameters are heart beat in beat per minute (bpm), peak velocity (cm/s), average velocity (cm/s), and ejection time (ms). Heart beat is calculated by identifying number of peaks in a known time duration. Software enables identifying peak forward velocity (shown as PFV in Figure 5), start of forward velocity (shown as FVS in Figure 5), and end of forward velocity (shown as FVE in Figure 5) for each beat in the spectrogram. Ejection time is the time from FVS to FVE and represents the time duration where the valve is open. Peak velocity is calculated by averaging all peak velocities in the spectrogram. Average velocity is the average of velocity averages for each beat. For Indus software, these can be calculated directly by the software. The envelope can be exported as a data file to Microsoft Excel or other similar program to plot velocity profiles.

In the following sections, adaptation of the Indus mouse Doppler platform for chick embryo and zebrafish studies are explained in detail. We will first give a brief review on the use of these animal models in cardiac research and then will describe our approach in using the Indus Doppler system to assess cardiac function by demonstrating representative data.

## Adaptation of the Mice Doppler System for Embryonic Chick Flow Measurements

Chick embryos are often used as a biological model of cardiac development. The model offers several advantages, for example the embryo develops in a planar orientation on top of the egg yolk, enabling a variety of imaging and local microsurgical options to alter blood flow. Furthermore, the cardiogenic period of the chick is longer than other species enabling more detailed spatiotemporal analysis. Another major advantage is that chick can adapt to microsurgical treatments well and are of limited ethical concern. Finally, the chick embryonic heart develops similarly to the human embryonic heart, with four chamber four valve configuration (Midgett and Rugonyi, 2014). Owing to those features, embryonic chick is an ideal model to study development of CHDs under abnormal hemodynamics.

In a developing embryo, heart is the first organ that starts to function. It facilitates embryonic growth as it converts nutrients to surrounding tissues. The heart starts as a linear valve-less tube. As the embryo grows, it transforms to a multi-chambered structure that comprises of four chambers and four fibrous valves in higher species (Srivastava and Olson, 2000; Beis et al., 2005; Butcher et al., 2007; England et al., 2016). Despite some differences with human heart, for example during septation and aortic arch remodeling, avian heart resembles the human anatomy (Supplementary Figure 1) more closely than other non-mammalian models (Andersen et al., 2014). It was originally thought that the sole function of a beating heart during the embryonic development is pumping blood for convective transport of blood throughout the body. However, it was later shown that, diffusion is a sufficient means of transport for oxygen, nutrients, metabolic wastes, and hormones in the early embryo (Burggren, 2004). On the other hand, mechanical perturbation of blood flow causes abnormal cardiogenesis, suggesting hemodynamic forces generated by contraction of cardiomyocytes in fact act to drive cardiogenesis (Granados-Riveron and Brook, 2012; Samsa et al., 2013; Lindsey et al., 2014). During normal development, flowing blood exerts several forces on surrounding tissue. These forces include the blood pressure force on the walls, the associated circumferential stress that occurs as the walls stretch in response to pressure, and the frictional force exerted by flow along the walls, wall shear stress (Gjorevski and Nelson, 2010). These mechanical signals induce gene expression and differentiation on a cellular level, translating molecular level events into tissue-level formations that guide embryo development (Wang et al., 2009; Mammoto and Ingber, 2010; Yalcin et al., 2011; Bharadwaj et al., 2012; Buskohl et al., 2012). Therefore, disturbed hemodynamics is a major epigenetic source for congenital heart defects.

Disturbing the hemodynamics by altering blood flow in chick embryo can easily be established through surgical intervention. The micro-surgery approaches are to constrict blood flow at certain locations in the heart to recreate a hemodynamically driven clinical CHDs. A good example of inducing CHD in chick embryo is left atrial ligation (LAL). LAL is a surgical approach for studying the development of hypoplastic left heart syndrome (HLHS). In LAL, a suture is placed around the left atrium and tied in a knot to constrict the left atrioventricular (AV) orifice and to decrease the effective volume of the left atrium (Yalcin et al., 2010; Midgett and Rugonyi, 2014). LAL has been performed at day 3–4, during the looping stages and before septation (Tobita and Keller, 2000; Tobita et al., 2002; Lucitti et al., 2005; Hu et al., 2009). The partial ligation of the left atrium reduces its size, narrows the inflow area of the left ventricle (LV), and redirects blood flow from the left to the right side of the heart. The redistributed hemodynamic load results in the hypoplasia (underdevelopment) in the left side and hyperplasia (overdevelopment) in right side cardiac structures (Tobita and Keller, 2000). Since the left side hypoplasia is a characteristic of HLHS, phenotype generated via LAL is accepted as an animal model of human HLHS (Midgett and Rugonyi, 2014). Other surgical interfaces in chick embryo include vitelline vein ligation (VVL) which is a process in which one of the vitelline veins that drain blood to the embryonic heart is ligated or clipped and conotruncal banding (CTB) where the outflow tract (OFT) is narrowed with a suture (Lucitti et al., 2005; Pang et al., 2017) . Similar to LAL, also these microsurgeries are usually performed on embryonic day 3–4, since heart at this stage is very sensitive to blood flow alterations (Hove et al., 2003). All of these microsurgeries produce specific phenotypes resembling different human CHDs. These disease models help to investigate the disturbed hemodynamics during disease progression for understanding the etiology as well as for generating new therapeutic approaches.

These surgical procedures require direct access to the embryo. This can be managed by culturing the chick embryos either outside their shells (*ex-ovo*) or within their shells (*in-ovo*). *Ex-ovo* culture requires yolk and the embryo to a culture platform such as a petri dish, or a hammock like structure (Yalcin et al., 2010). *In-ovo* culturing method requires opening of a small window on the shell. For both techniques, egg shell should be cut at a stage where the vitelline vessels are not attached to the walls. Also, sufficient time should be given to the embryo to develop inside the shell. In our practice, we found out that, day 3 is the optimal time to crack the egg for *ex-ovo* culture, or open the egg window for *in-ovo* culture. For both cultures, external environmental interferences have to be minimized. In here, we use the *in-ovo* culture system to measure blood flow velocities using the mice Doppler platform.

### *In-ovo* Chick Embryo Culture

**Figure 6:**
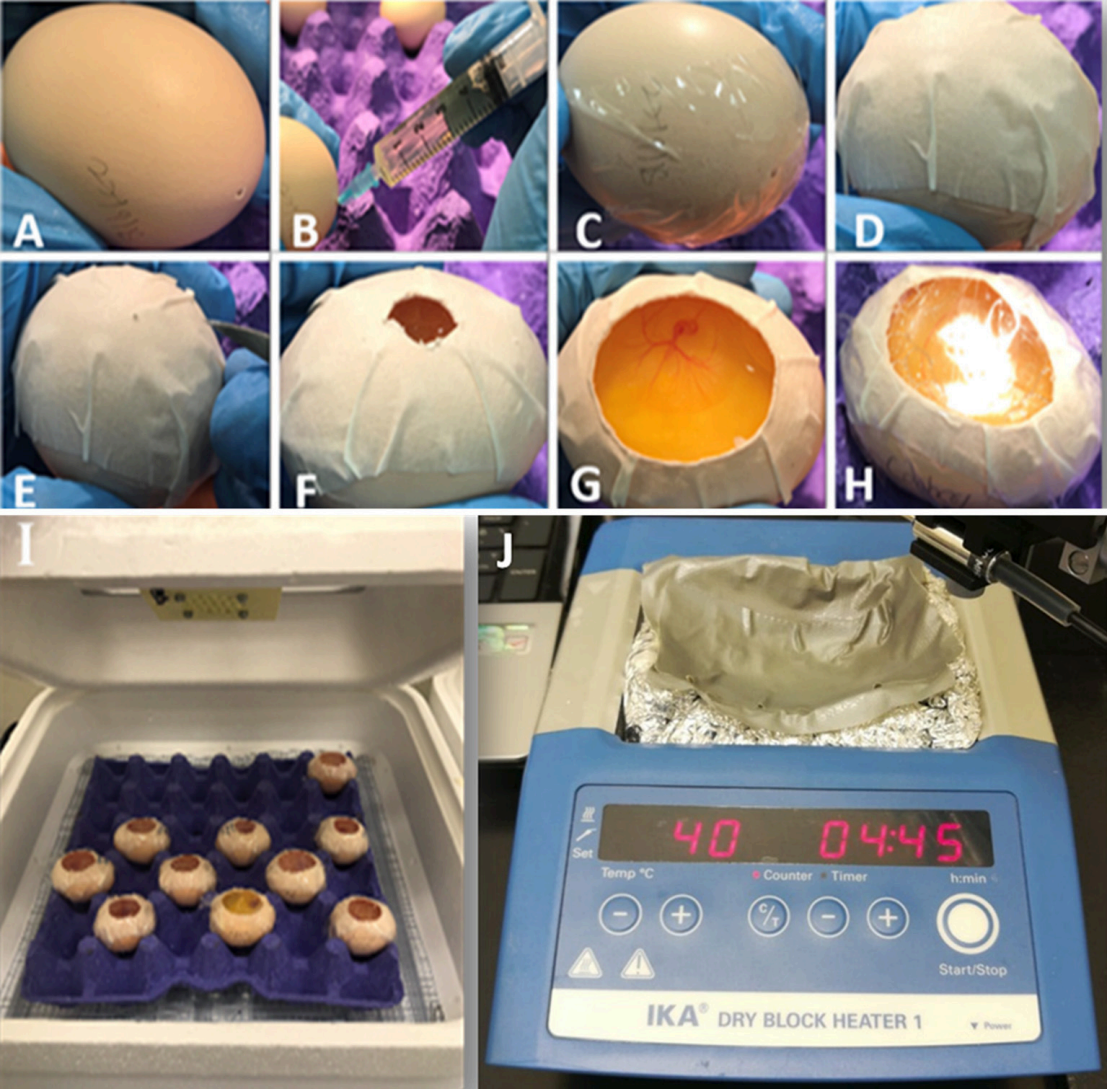
Details of *in-ovo* culture and temperature stabilization during Doppler measurements. **(A)** The egg is kept horizontal for few minutes. **(B)** A hole is made using dissection scissors and with the aid of a needle and a syringe about 5 ml of the albumin is removed. **(C)** A clear tape is used to cover the hole. **(D)** Paper tape is placed on the top horizontal side to stabilize the egg. **(E)** Using dissection scissors, a small hole. **(F)** The hole is enlarged to locate the embryo. **(G)** Once the embryo is located, the hole is enlarged to allow access to the embryo. **(H)** Using a clear tape, the window is covered. **(I)** Eggs are then returned to the incubator. **(J)** Temperature stabilization for chick embryo using dry block heater covered by aluminum foil containing armor beads. The setup is sealed with water resistant tape. Water is added on top of the tape and to reach a water temperature of 37°C the heat block should be placed at 40°C.

Fertilized eggs should be incubated directly after laying in a 37.5°C incubator with 60% humidity; however, if there is a requirement to delay the experiments, eggs can be reserved up to 5–7 days in a 13°C cooler before the embryos start development. On day 0, eggs are placed blunt end to the top in an incubator, with continuous rocking for 72 h (3 days). On day 3, the eggs are taken out of the incubator in batches of 10 so that their temperature does not drop drastically. They are then kept laying horizontally for few minutes to allow the embryo to relocate to the top of the egg. On the blunt end, a hole is gently made with a surgical scissor. While the egg is stable, a 19-gauge needle attached to a 5 ml syringe is inserted vertically inside the egg, with caution not to poke the yolk, to remove about 5 ml of the albumin (egg white). This is done to lower the yolk with embryo for preventing rupture of the yolk with scissor penetration during cutting. The hole is then covered by clear adhesive tape. White paper tape is placed to the top of the egg to facilitate opening a widow without harming the embryo. The widow is made by creating a hole then enlarging it by cutting in a spiral form. Finally, created window is covered by a transparent tape and eggs are placed in a portable incubator under the same conditions. These steps are illustrated in Figures 6A–I.

### Embryo Environmental Stabilization

Since the procedure of measuring blood hemodynamics is performed outside the incubator, the fluctuating temperature will almost certainly interfere with the recorded data and create variabilities across the study groups. As such, embryos must be maintained at the same temperature that they were incubated at to minimize environmental interferences. We have developed an easy and affordable setup that maintains the embryos at a temperature of 37°C during analysis. The setup requires a dry block heater, lab Armor beads, aluminum foil, water resistant tape, and water. The aluminum foil is placed in the dry block then filled with the Armor beads. The beads are then covered with foil and a groove that resembles the shape of the egg is made. These beads are good heat conductors, however to assure good conduction through the egg, the groove is covered with the water resist tape and a small amount of water is added to the groove. This way, heated water would conduct the heat through contact with the egg. For this particular setup (Figure 6J), we found that setting the heat block to 40°C results in a water temperature of 37°C. Once the water temperature reaches 37°C, the eggs can be placed on top of the shallow water and the experiment can be started.

### Chick Embryo Blood Flow Velocity Measurement Using the Mice Doppler System

To access the embryo, the chorionic and allantoic membranes need to be removed using a dissecting stereo microscope and a pair of sharp forceps to expose the heart. For Doppler measurements, eggs are placed on the preheated dry block setup. Few drops of prewarmed Tyrode's solution is applied on the embryo near the heart to couple sound waves. The embryo's orientation is crucial and it should be set exactly as described below to reduce the angle between the probe and the detected flow to zero degrees, so that sound waves and blood flow are fully aligned. Furthermore, the channel being used in the PDT should be set to “AWAY” as the flow direction is moving away from the probe, which will result in getting positive velocity values since blood is moving away from the probe.

**Figure 7:**
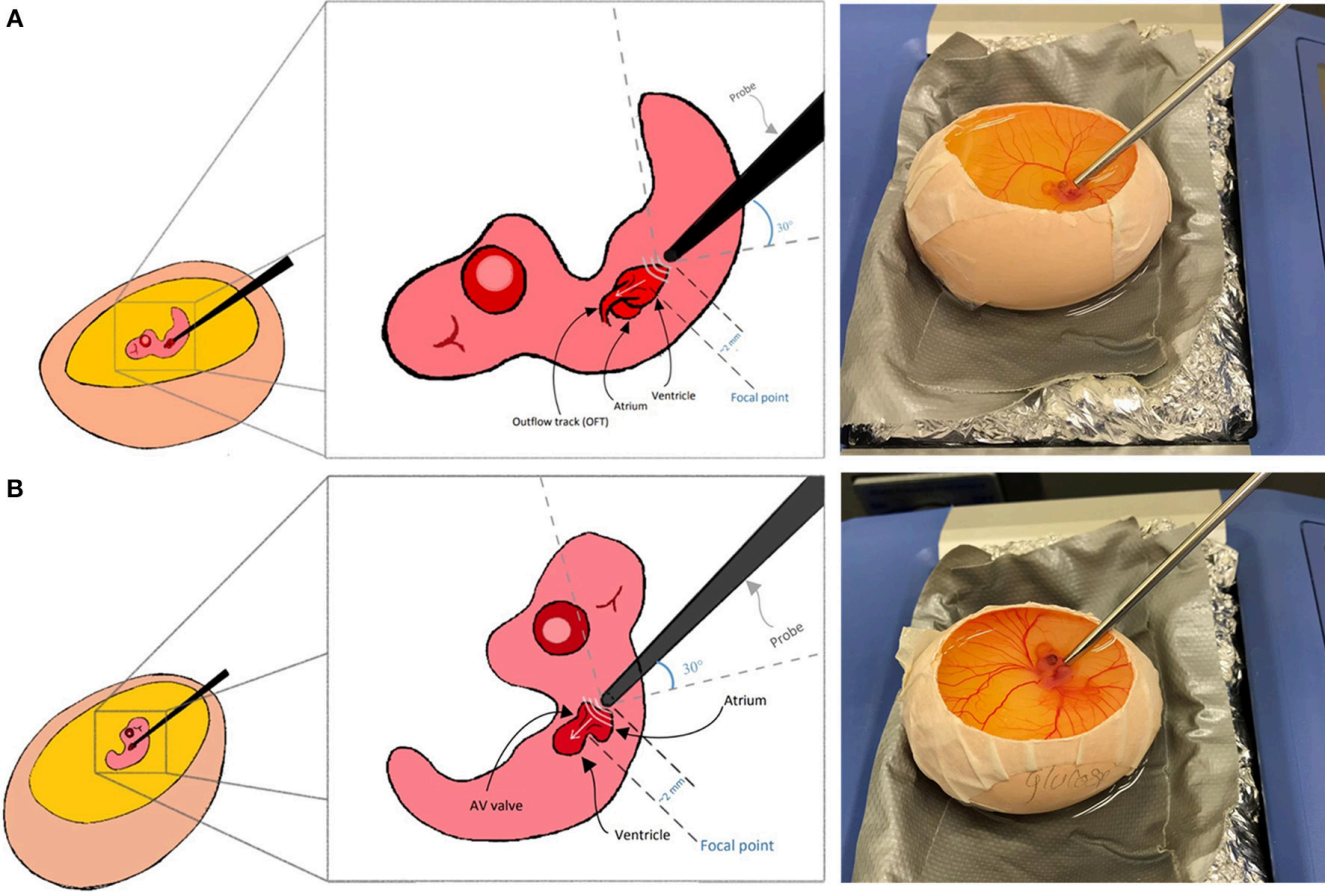
Chick embryo orientation for Doppler echocardiography analysis. **(A)** For outflow track blood flow measurement, the embryo is kept laying on its left side and the transducer probe is oriented as shown in the figure. **(B)** For blood flow measurement at the atrioventricular valve, embryo should be gently flipped using a blunt forceps and the transducer probe should be oriented as shown in the figure.

Here we explain Doppler blood flow measurements for embryonic day 5 as an example. This is a pre-septation stage where the heart is composed of one main ventricle, one atria, one AV valve, and one OFT valve. AV and OFT valve measurements are presented here. For other stages, minor adjustments to below protocol may be needed. A 20 MHz probe is suitable for this application. The chick embryo naturally lays on its left side, exposing the right side on top. To get good signal from the OFT, we found that, the natural orientation of the embryo is good. The probe is oriented toward the OFT valve from the apex, from the embryo‘s tail side. At that configuration, the probe is oriented at angle of around 30 degrees with the horizontal surface as shown in Figure 7A. To get the signal from the AV valve, we found that, Doppler transducer needs to approach the embryo from its left side on top. Therefore, the embryo is gently flipped with a blunt forceps and the probe is placed between the atria and the ventricle just near the head where the eye is located as shown in Figure 7B. For AV valve, the probe is again oriented at about 30 degrees to the horizontal surface. Doppler velocity signal acquisition was explained above and hence is not repeated here. It is appropriate to save a signal for about 5 s which will save about 10–15 peaks, sufficient for further analysis.

**Figure 8:**
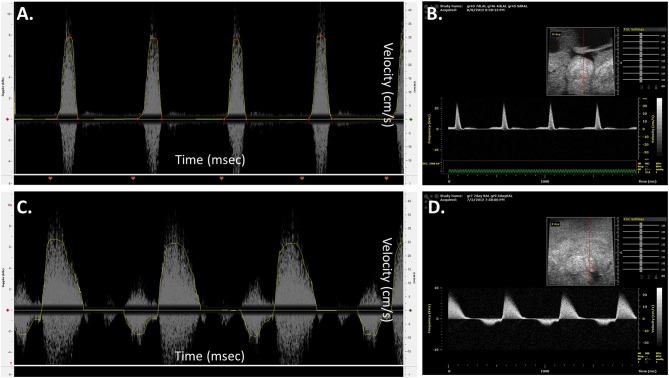
Chick embryo blood flow velocity waveforms obtained by mice Doppler system **(A,C)** and Visualsonics *in vivo* 770 platform **(B,D)**. The waveforms were measured at the atrioventricular **(A,B)** and outflow track canals **(C,D)**. The data obtained by both platforms compares well. The spectrograms from the mice Doppler system **(A,C)** shows a mirroring shadow of the recorded signal. The analysis software, however, recognizes this and detects only the correct signal, traced in a yellow line.

**Figure 9:**
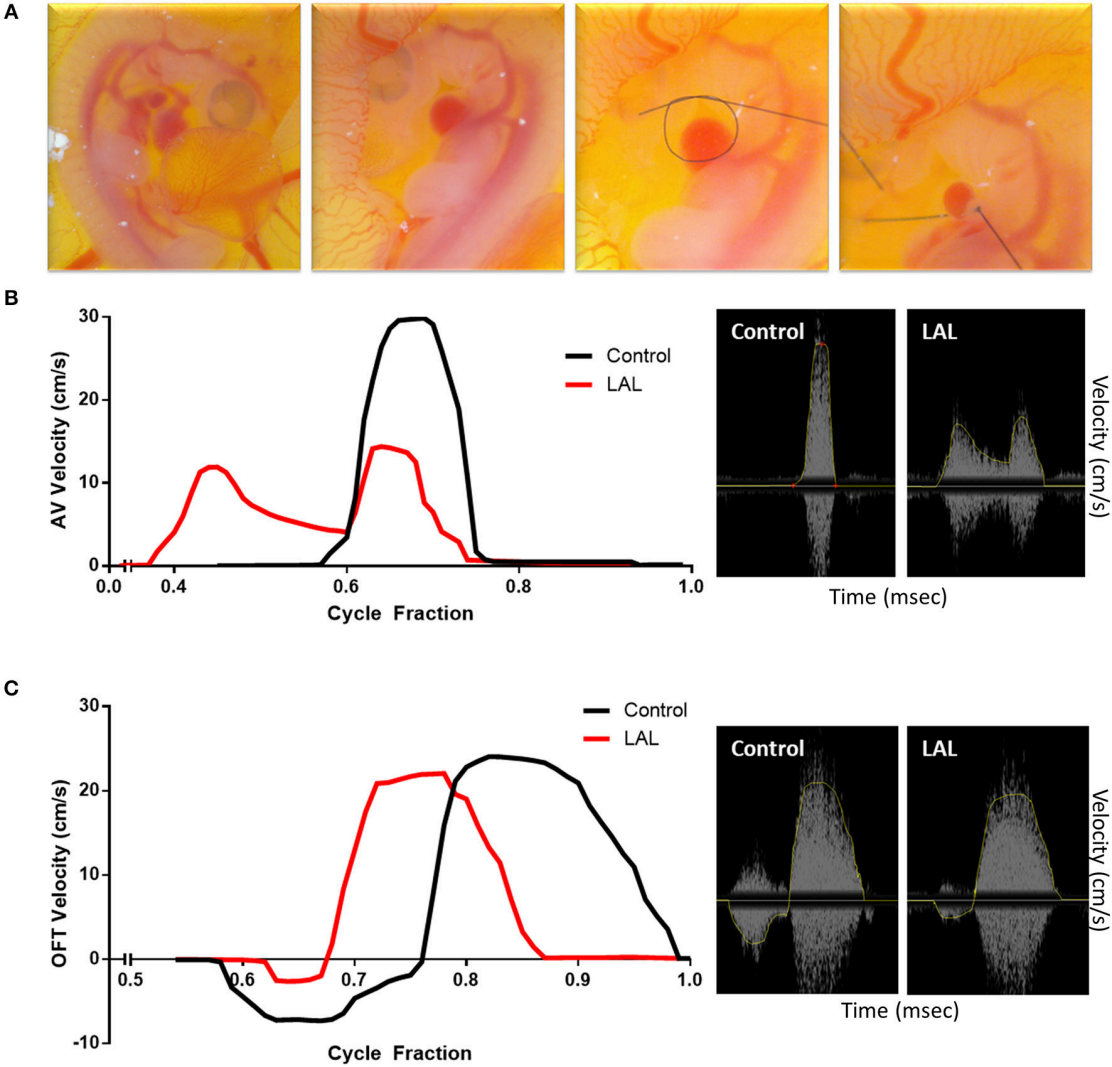
Effect of left atrial ligation (LAL) on blood flow velocity. **(A)** Steps of LAL procedure; the embryo sits on its left side, it is flipped to have access to the left atria (LA). A pre-prepared knot using 10-0 nylon surgical suture is positioned over the left atrium then tightened so that the volume of the LA is reduced to about 75%. Extracted data of blood flow velocities at the **(B)** atrioventricular (AV) and **(C)** outflow track (OFT) canals over cycle fraction along with an example waveform peak for LAL and control embryos. The velocity profile at the AV canal of the LAL group had a wider spread with higher passive contraction peak (first peak) and lower active contraction peak (second peak). The velocity profile at the OFT canal of the control groups showed an initial regurgitating flow whereas the LAL group OFT canal velocity profile did not show this behavior.

Prior to proceeding with analyzing diseased models, blood flow velocities of control embryos were measured and compared with published data obtained using different and more advanced platforms. Figures 8B,D illustrates velocity profiles that were extracted previously using Vevo 770 echocardiography platform for normal day 5 embryos (Yalcin et al., 2011). The data obtained from the mice Doppler system (Figures 8A,C) on the same day compares well. Spectrograms that represents blood flow velocities at the AV (Figure 8A Mice Doppler system and Figure 8B previous data) and OFT valves (Figure 8C Mice Doppler system and Figure 8D previous data) shows that the waveforms as well as the velocity peaks extracted from both platformers measurements are similar. The difference between advanced platforms and the mouse Doppler system is the appearance of a shadow in the spectrogram for the Indus system. The shadow is a mirror image of the recorded waveform with a lower brightness. However, the software recognizes this and only detects the actual signal. The velocity envelope can also be further corrected by the user with that information. Figures 9B,C, illustrates extracted waveforms over one cardiac cycle for blood flow velocities at the AV and the OFT canals. The reproducibility of the blood flow velocity waveforms and good comparison with the data obtained by advanced platforms validates the Indus platform as a good tool for blood flow velocities measurement in the chick embryo.

### Effect of Left Atrial Ligation (LAL) Microsurgical Procedure on the Heart's Hemodynamics

To further evaluate the system for embryonic chick cardiac disease models, we studied velocity profiles after LAL. As mentioned earlier, LAL is a microsurgery where a nylon knot suture is tied around the left atrium of the heart. LAL was performed on the 4th day of incubation, during cardiac looping but ahead of septation. Figure 9A, summarizes the microsurgical steps. Briefly, the chorionic and allantoic membranes are removed over the embryo grown in our *in-ovo* culture. Naturally, the embryo sits on its left side; therefore, the embryo is lifted from the back and vertically rotated in order to access the left heart. Pericardium over the left atria (LA) is then cut and removed with fine forceps. Knots of approximately 0.5 mm diameters are prepared from 10-0 nylon surgical suture. These knots are aligned over the LA then tightened so that the volume of the LA is reduced to about 75%. This interference is expected to constrict the blood flow through the left side of the AV canal. The embryo is rotated back, to its original position following the procedure so that the right side is on top. Details of this procedure can be seen in our video protocol (Yalcin et al., 2010).

**Figure 10:**
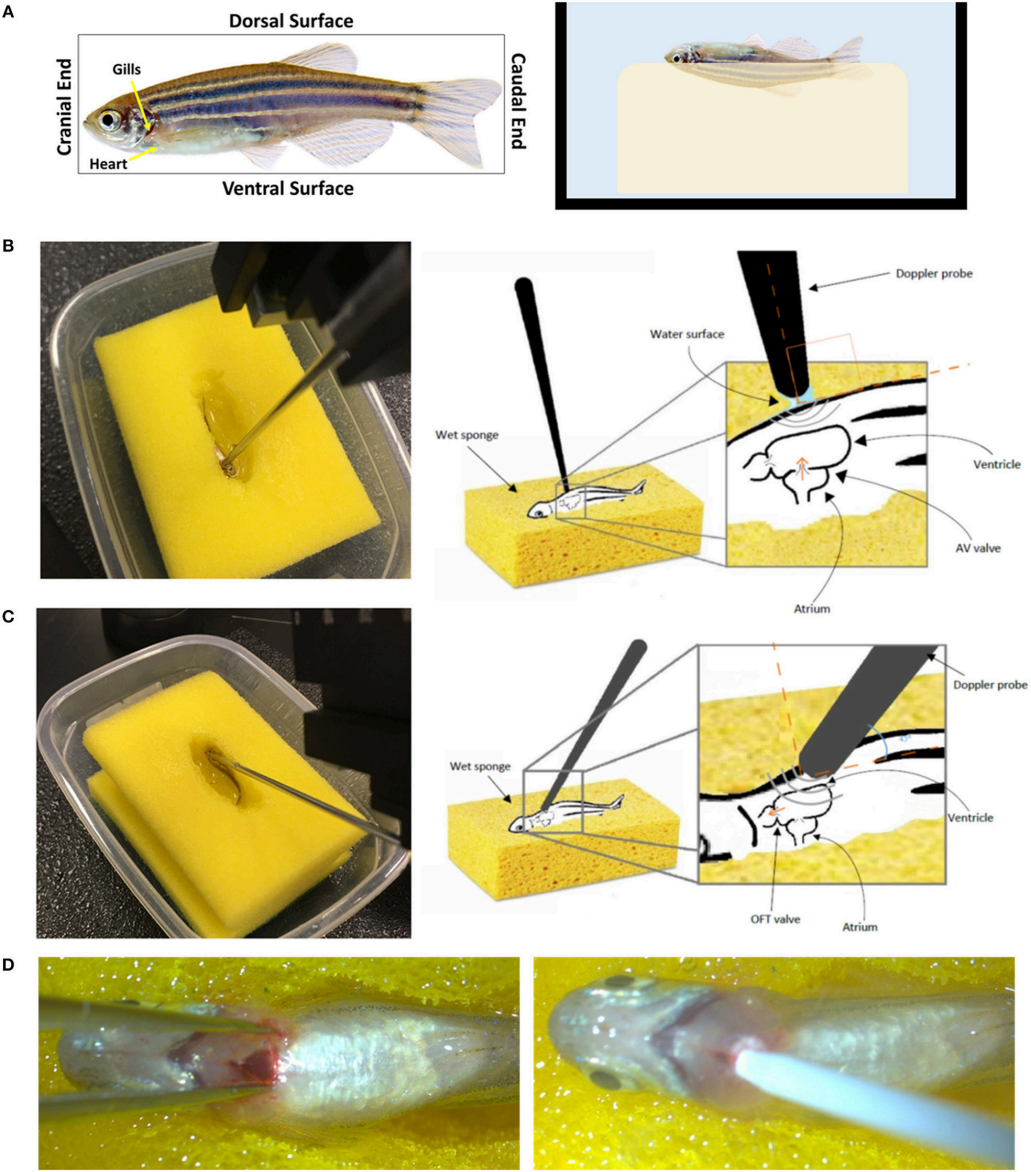
Setup and orientation of zebrafish for Doppler echocardiography analysis. **(A)** The fish is stabilized on a wet sponge on its dorsal side toward the sponge making sure it is fully immersed in water. The 20 MHz transducer probe is positioned **(B)** perpendicular to the ventral side of the fish to obtain the atrioventricular valve (AV) flow or **(C)** at an angle to obtain the outflow rack (OFT) flow. **(D)** Myocardial infarction induction using cryoinjury. The fish in **(A)** was obtained from www.shutterstock.com.

The changes in the hemodynamics at the AV and OFT canals was assessed 24 h following LAL. Figures 9A, 10B shows the velocity profiles at the AV and OFT canals, respectively, over cardiac cycle for control as well as for LAL hearts. The velocity profile at the AV canal of the control groups showed distinct peaks representing passive (first peak) and active (second peak) contractions. On the other hand, the velocity profile at the AV canal of the LAL group had a wider spread with higher passive contraction peak and lower active contraction peak. For the OFT canal, the velocity profile of the control groups showed an initial regurgitating flow whereas the LAL group OFT canal velocity profile did not show this behavior. Both AV and OFT velocities match very well with our previous measurements with Vevo 770 system. More specifically, In AV channel, we see a dramatic decrease in peak velocity, suggesting decrease in WSS levels. However, average velocity does not differ in AV, suggesting preservation of cardiac output. In OFT no significant change is observed rather than disappearance of initial regurgitation for LAL embryos. Tabulated data can be found in Table 1. These changes were expected after LAL surgery (Yalcin et al., 2011), which again validates the mice Doppler system blood flow measurements.

**Table 1:** Day 5 chick embryo heart rate and blood flow velocities that were obtained with Indus Doppler system.

**Parameters**	**Control**		**LAL**		***P*****-value**	
Heart Rate (bmp)	166.45 ± 5.11		120.83 ± 1.69		<0.0001	
**Blood flow velocity**	**AV**			**OFT**		
	**Control**	**LAL**	***P*****-value**	**Control**	**LAL**	***P-*****value**
Peak velocity (cm/s)	30.08 ± 0.72	15 ± 0.74	< 0.0001	23.62 ± 1.40	23.38 ± 0.77	>0.9999
Ejection time (ms)	98.6 ± 4.63	261.8 ± 6.62	< 0.0001	181.5 ± 3.25	208.29 ± 6.88	< 0.0001
Mean velocity (cm/s)	4.48 ± 0.10	4.42 ± 0.13	>0.9999	5.99 ± 0.58	6.29 ± 0.25	>0.9999

*Data is presented as mean ± SD. Analysis was by unpaired student t test. A p < 0.05 was considered as statistically significant. A total of 5 embryos were analyzed per group*.

## Adaptation of Mice Doppler System for Adult Zebrafish Flow Measurements

During the past two decades, zebrafish, *Danio rerio*, a small tropical freshwater fish, has become a popular vertebrate model for research. This was mainly due to the number of large-scale mutagenesis screens that have been conducted successfully with this animal model (Kari et al., 2007). Its high similarity to human gene sequence and function (87% similarity) may indicate why zebrafish can be used to model human diseases (Lieschke and Currie, 2007; Howe et al., 2013). It is estimated that 70% of the human genes have ortholog genes in the zebrafish genome (Barbazuk et al., 2000; Bournele and Beis, 2016). Compared to other mammalian models, zebrafish offer several advantages such as rapid development and reproduction, convenient genetic manipulation techniques, and low cost maintenance. These criteria rendered this small fish as an ideal organism to study the genetic basis of disease. A single female can produce ~200 eggs weekly allowing for large scale analysis (Parng et al., 2002; Poon and Brand, 2013). Furthermore, the zebrafish embryos are fertilized externally allowing for quick collection and genetic manipulation (Miura and Yelon, 2011).

Even though zebrafish does not spontaneously develop cardiovascular disorders analogous to humans (McLeish et al., 2010), a number of conditions can be readily modeled for cardiac research. Several cardiovascular specific transgenic strains have been generated. Zebrafish have a cardiovascular system with a tubular two-chambered heart with gills instead of lungs. The heart develops early during embryogenesis, and the heart starts to beat at 24 hpf (hours post fertilization). Supplementary Figure 2 demonstrates the stages of zebrafish heart development and compares an adult zebrafish heart to a human heart. The anatomical differences between zebrafish and humans are considerable, but the ease of studying zebrafish and rapid rate of cardiac development makes them a valuable model for heart disease. The zebrafish cardiomyocyte action potential seems almost identical to their humans' counterparts (Verkerk and Remme, 2012). Cardiac related diseases that are currently studied in zebrafish include congenital heart diseases, heart failure, cardiomyopathy, cardiac arrhythmia, myocardial infarction, and valvular heart disease (Chi et al., 2008; Bakkers, 2011; Dhillon et al., 2013; Asnani and Peterson, 2014; Liu et al., 2016). Since zebrafish heart develops rapidly, cardiac drug screening in the zebrafish can be performed early, but is likely best done after 96 hpf. Studying how cardiac function is affected by genetic manipulation, drug or toxin exposure, or cardiac intervention in zebrafish may help to both understand the mechanism of action and reveal new therapeutic targets. Time-lapse microscopy is sufficient to study cardiac function in zebrafish embryos and larvae, since zebrafish have transparent skin at early stages, enabling the direct visualization of the heart and blood vessels. For cardiovascular assessment for zebrafish embryo/larvae, detailed protocols can be found in our previous work (Yalcin et al., 2017; Eisa-Beygi et al., 2018; Zakaria et al., 2018). As the zebrafish age and lose skin transparency the Doppler technique must be used for heart function assessment. We have also adapted a Doppler system used for mice as a tool to measure the heart valve blood flow velocities in adult zebrafish. Below is a description of our method.

### Stabilization of Adult Zebrafish

Adult fish need to be immobilized to allow cardiac measurements. This can be done by anesthetization prior to the procedure. Tricaine methanesulfonate (Tricaine) is a good agent that is commonly used to sedate the fish (Carter et al., 2011). For Doppler analysis, the fish is transferred to a tank containing a final concentration of 90 mg/L Tricaine, prepared in water obtained from the fish system, for several minutes until the fish loses equilibrium and spinal reflexes. As mentioned previously, orientation is crucial when it comes to Doppler analysis, this presents a challenge when attempting to observe free-floating fish. A simple method to keep the fish in the correct orientation and in a wet environment is using a water soaked sponge. A small hole that resembles the shape of the fish is made in the middle of a sponge. The sponge is put in a tray then soaked with water from the fish system. The sponge has to be saturated with water so that the created hole is filled with water. The fish is then moved to the hole and Doppler analysis can be initiated. It is important that the fish is maintained at 25–29°C during the analysis as abnormal temperatures will interfere with cardiac measurement.

### Adult Blood Flow Velocity Measurement Using the Mice Doppler System

The procedure has to be performed relatively quickly before the anesthesia wears of. Once the fish starts to flick its tail, the experiment has to be stopped and the fish has to be transferred to a tank of fresh system water to recover. Anesthetizing fish in 90 mg/L tricaine allows for a 5-min window for Doppler analysis. The breathing of the fish needs to be closely monitored during the measurement. This can be done through examining the gills. If any changes in the base line of the movement of the gills is observed, the experiment has to be stopped and the fish needs to be put back the fresh system water to recover.

The fish has to be oriented with its dorsal surface toward the sponge and its ventral surface facing upwards (Figure 10A). This orientation has been previously described when measuring cardiac function using more advanced platforms (Hein et al., 2015; Wang et al., 2017). It is unnatural to the fish and it might induce stress, consequently affecting the heart rhythm and cardiac output. This is a limitation about the animal model since all imaging modalities, including the one described here, require stabilizing the fish in such way to have access to the heart.

To measure the blood flow velocities at the AV valve, the probe should be perpendicular on the fish, toward the cranial end, and just below the gills (Figure 10B). To measure the blood flow velocities at the OFT valve, the angle of the probe has to be adjusted to 45 degree with the fish's horizontal axes. The probe's head has to be oriented toward the cranial end and the base toward the caudal end (Figure 10C). These orientations will assure ultrasound signals and blood flow direction are fully aligned (Instruments, 2017). The water around the fish creates an aqueous contact zone between the ultrasound probe and the animal, whereby up to 1 cm standoff can be maintained via liquid surface tension. Once the procedure is completed, the fish has to be immediately placed in a tank full of system water for recovery.

**Figure 11:**
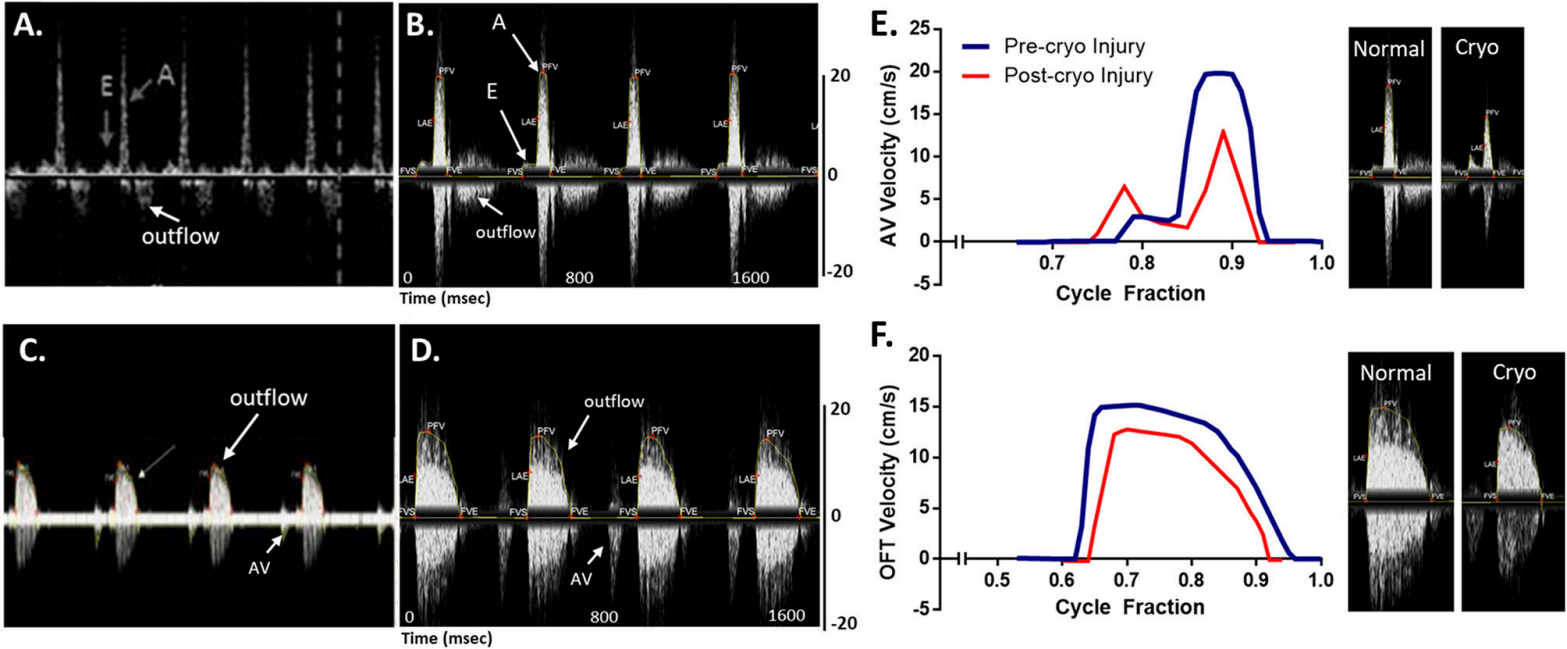
Adult zebrafish blood flow velocity waveforms. **(A,C)** Waveforms obtained from literature (Instruments, 2017). **(B,D)** Waveforms obtained by the Indus mice Doppler system. **(A**,**B)** The waveforms were measured at the atrioventricular. **(C,D)** The waveforms were measured at the outflow track canals. Both set of data compare well, showing the same peaks with the same velocity profile. **(E,F)** Extracted data of blood flow velocities. Data was obtained from control and myocardial infarcted adult zebrafish at the **(A)** atrioventricular (AV) and **(B)** outflow track (OFT) canals over cycle fraction along with example waveform peaks.

Blood flow velocities were measured at the AV and OFT valves for normal wild type (AB) zebrafish at 1 year of age. A 20 MHz Doppler probe transducer was used for this purpose. Previous Doppler echocardiography examinations on adult zebrafish showed distinct E and A waves in AV velocity profile, representing filling of the ventricle at early diastolic inflow (E) and late diastolic inflow (A) (Lee et al., 2014; Hein et al., 2015; Instruments, 2017; Packard et al., 2017). Negative OFT velocities are also detected while measuring AV velocity, as seen in Figure 11A. OFT velocity profile obtained from a previous study is shown in Figure 11C. These two velocity profiles were used as a reference for the mice Doppler measurements to confirm correct readings. For the AV profile using Indus Doppler system (Figure 11B), distinct E and A waves were also seen in the image, where E wave was smaller than A wave. Similar to previous studies, the OFT velocity signal was also present in the AV recording. The OFT velocity profile using Indus Doppler system (Figure 11D), identical to previous measurements, included a wider region of positive velocities. The heartbeat and other blood flow velocities are summarized in Table 2. From AV valve velocity profile, measuring E and A waves velocities are used to calculate E/A ratio. This ratio is used to assess diastolic function. Unlike human, zebrafish E/A ratio is smaller than one (Packard et al., 2017). In zebrafish cardiomyopathy and myocardial infarction models, this ratio was shown to increase (Lee et al., 2014; Hein et al., 2015; Packard et al., 2017) which suggests ventricular diastolic dysfunction. Figures 11E,F (blue line), illustrates extracted waveforms of the blood flow velocities at the AV and OFT canals over the cycle fraction.

**Table 2:** Adult zebrafish heart rate and blood flow velocities that were obtained with Indus Doppler system.

**Parameters**	**Control (Pre cryo)**		**Post cryo**		***P*****-value**	
Heart Rate (bmp)	121.95 ± 3.70		117.08 ± 50.24		0.8427	
**Blood flow velocity**	**AV**			**OFT**		
	**Pre cryo**	**Post cryo**	***P*****-value**	**Pre cryo**	**Post cryo**	***P*****-value**
Peak velocity (cm/s)	26.31 ± 3.89	14.71 ± 2.36	0.0155	16.52 ± 3.89	14.91 ± 2.79	0.3701
Ejection time (ms)	149.91 ± 27.58	314.9 ± 134.6	0.1162	265.7 ± 48.99	144.33 ± 8.22	0.0376
Mean velocity (cm/s)	2.79 ± 0.60	1.88 ± 0.41	0.1849	3.85 ± 0.64	2.63 ± 0.48	0.0144

*Data is presented as mean ± SD. Analysis was by paired student t test. A p < 0.05 was considered as statistically significant. A total of 3 adult fish were analyzed per group. Cryo, Cryoinjury*.

### Effect of Myocardial Infarction on the Zebrafish Heart's Hemodynamics

To further evaluate the system for zebrafish cardiac disease models, we studied velocity profiles after cardiac injury. Myocardial infarction was induced using cryoinjury method as described by Chablais and Jazwinska (2012). Briefly, a stainless steel rode (cryoprobe) with a diameter of 0.8 mm was cooled down by immersing the tip in liquid nitrogen for 3 min. Zebrafish was anesthetized using 90 mg/L tricaine. The fish was then stabilized as described above. Under a stereomicroscope and using a dissecting scissors and a sharp forceps, an incision was made just below the gills to expose the heart. The cryoprobe was obtained and access liquid nitrogen was removed by shaking the probe for 10 s. The incision was spread laterally using forceps and the heart's vertical was touched gently with the cryoprobe (Figure 10D). After 24 s, 2 ml of system water was added on to the surgical site to release the cryoprobe. The fish was immediate transferred to fresh system water to recover.

The changes in the hemodynamics at the AV and OFT canals was assessed 24 h following myocardial infarction. Figures 11E,F (red line) shows the velocity profiles at the AV and OFT canals, respectively, over cardiac cycle for control as well as for infarcted hearts. The velocity profile at the AV canal of the control groups showed distinct A peak and a smaller E peak. Myocardial infarction caused the A peak velocity to decrease and the E peak velocity to increase. These findings are comparable to that previously described (Lee et al., 2014; Hein et al., 2015; Packard et al., 2017). Tabulated data can be found in Table 2. These changes were expected after myocardial infarction, which validates the mice Doppler system for zebrafish blood flow measurements.

## Conclusions

Doppler echocardiography is an important and powerful tool for the assessment of cardiovascular physiology and function; it plays a vital role in the comprehensive evaluation of cardiovascular system particularly in the fetal stage of life. There are several echocardiography devices that measure cardiovascular function for small animals, however, they are usually designed for rodents, including rats and mice, and due to their complexity, they come with a high cost. Here we have adapted a single mode mice Doppler echocardiography system to measure cardiac flow velocities for adult zebrafish and embryonic chickens, and successfully assessed cardiac function for normal and diseased embryonic chicken and zebrafish. We believe that our presented approach will help cardiac researchers to establish similar echocardiography platforms in their labs in a practical and economical manner.

## Ethics Statement

All experiments presented in this paper were conducted under the Qatar University's Institutional Animal Care and Use Committee (QU-IACUC) approval.

## Author Contributions

FB wrote the manuscript. FB, MA, AS, and HA contributed to running the chick embryo experiments. FB, MA, and ZZ contributed to running the zebrafish experiments. HY supervised the studies and reviewed the manuscript. All authors read the manuscript.

### Conflict of Interest Statement

The authors declare that the research was conducted in the absence of any commercial or financial relationships that could be construed as a potential conflict of interest.

## References

[B1] Amindari A. Saltik L. Kirkkopru K. Yacoub M. Yalcin H. C. (2017). Assessment of calcified aortic valve leaflet deformations and blood flow dynamics using fluid-structure interaction modeling. Inform. Med. Unlocked. 9, 191–199. 10.1016/j.imu.2017.09.001

[B2] Andersen T. A. Troelsen Kde L. Larsen L. A. (2014). Of mice and men: molecular genetics of congenital heart disease. Cell. Mol. Life Sci. 71, 1327–1352. 10.1007/s00018-013-1430-1 23934094 PMC3958813

[B3] Asnani A. Peterson R. T. (2014). The zebrafish as a tool to identify novel therapies for human cardiovascular disease. Dis. Models Mech. 7, 763–767. 10.1242/dmm.016170 24973746 PMC4073266

[B4] Bakkers J. (2011). Zebrafish as a model to study cardiac development and human cardiac disease. Cardiovasc. Res. 91, 279–288. 10.1093/cvr/cvr098 21602174 PMC3125074

[B5] Balachandran K. Sucosky P. Yoganathan A. P. (2011). Hemodynamics and mechanobiology of aortic valve inflammation and calcification. Int. J. Inflam. 2011:263870. 10.4061/2011/263870 21760982 PMC3133012

[B6] Barbazuk W. B. Korf I. Kadavi C. Heyen J. Tate S. Wun E.. (2000). The syntenic relationship of the zebrafish and human genomes. Genome Res. 10, 1351–1358. 10.1101/gr.144700 10984453 PMC310919

[B7] Beis D. Bartman T. Jin S. W. Scott I. C. DAmico L. A. Ober E. A.. (2005). Genetic and cellular analyses of zebrafish atrioventricular cushion and valve development. Development 132, 4193–4204. 10.1242/dev.01970 16107477

[B8] Bharadwaj K. N. Spitz C. Shekhar A. Yalcin H. C. Butcher J. T. (2012). Computational fluid dynamics of developing avian outflow tract heart valves. Ann. Biomed. Eng. 40, 2212–2227. 10.1007/s10439-012-0574-8 22535311 PMC3549459

[B9] Bournele D. Beis D. (2016). Zebrafish models of cardiovascular disease. Heart Fail. Rev. 21, 803–813. 10.1007/s10741-016-9579-y 27503203

[B10] Bruneau B. G. (2008). The developmental genetics of congenital heart disease. Nature 451, 943–948. 10.1038/nature06801 18288184

[B11] Burggren W. W. (2004). What is the purpose of the embryonic heart beat? Or how facts can ultimately prevail over physiological dogma. Physiol. Biochem. Zool. 77, 333–345. 10.1086/422230 15295688

[B12] Buskohl P. R. Jenkins J. T. Butcher J. T. (2012). Computational simulation of hemodynamic-driven growth and remodeling of embryonic atrioventricular valves. Biomech. Model. Mechanobiol. 11, 1205–1217. 10.1007/s10237-012-0424-5 22869343 PMC3536825

[B13] Butcher J. T. McQuinn T. C. Sedmera D. Turner D. Markwald R. R. (2007). Transitions in early embryonic atrioventricular valvular function correspond with changes in cushion biomechanics that are predictable by tissue composition. Circ. Res. 100, 1503–1511. 10.1161/CIRCRESAHA.107.148684 17478728

[B14] Camacho P. Fan H. Liu Z. He J. Q. (2016). Small mammalian animal models of heart disease. Am. J. Cardiovasc. Dis. 6, 70–80. 27679742 PMC5030387

[B15] Carter K. M. Woodley C. M. Brown R. S. (2011). A review of tricaine methanesulfonate for anesthesia of fish. Rev. Fish Biol. Fish. 21, 51–59. 10.1007/s11160-010-9188-0

[B16] Chablais F. Jazwinska A. (2012). Induction of myocardial infarction in adult zebrafish using cryoinjury. J. Vis. Exp. e3666. 10.3791/3666 22546770 PMC3466631

[B17] Chi N. C. Shaw R. M. Jungblut B. Huisken J. Ferrer T. Arnaout R.. (2008). Genetic and physiologic dissection of the vertebrate cardiac conduction system. PLoS Biol. 6:e109. 10.1371/journal.pbio.0060109 18479184 PMC2430899

[B18] Chorro F. J. Such-Belenguer L. Lopez-Merino V. (2009). Animal models of cardiovascular disease. Rev. Esp. Cardiol. 62, 69–84. 10.1016/S0300-8932(09)70023-5 19150017

[B19] Culver J. C. Dickinson M. E. (2010). The effects of hemodynamic force on embryonic development. Microcirculation 17, 164–178. 10.1111/j.1549-8719.2010.00025.x 20374481 PMC2927969

[B20] Dawson T. H. (1991). Engineering Design of the Cardiovascular System of Mammals. New Jersey, NJ: Prentice Hall.

[B21] Dhillon S. S. Dóró É. Magyary I. Egginton S. Sík A. Müller F. (2013). Optimisation of embryonic and larval ECG measurement in zebrafish for quantifying the effect of QT prolonging drugs. PLoS ONE 8:e60552. 10.1371/journal.pone.0060552 23579446 PMC3620317

[B22] Eisa-Beygi S. Benslimane F. M. El-Rass S. Prabhudesai S. Abdelrasool M. K. A. Simpson P. M.. (2018). Characterization of endothelial cilia distribution during cerebral-vascular development in zebrafish (*Danio rerio*). Arterioscler. Thromb. Vasc. Biol. 38, 2806–2818. 10.1161/ATVBAHA.118.311231 30571172 PMC6309420

[B23] England J. Pang K. L. Parnall M. Haig M. I. Loughna S. (2016). Cardiac troponin T is necessary for normal development in the embryonic chick heart. J. Anat. 229, 436–449. 10.1111/joa.12486 27194630 PMC4974548

[B24] Ernens I. Lumley A. I. Devaux Y. Wagner D. R. (2016). Use of coronary ultrasound imaging to evaluate ventricular function in adult zebrafish. Zebrafish 13, 477–480. 10.1089/zeb.2016.1274 27326768 PMC5124742

[B25] Fernández Esmerats J. Heath J. Jo H. (2016). Shear-sensitive genes in aortic valve endothelium. Antioxid. Redox Signal. 25, 401–414. 10.1089/ars.2015.6554 26651130 PMC5011632

[B26] Forouhar A. S. Liebling M. Hickerson A. Nasiraei-Moghaddam A. Tsai H. J. Hove J. R.. (2006). The embryonic vertebrate heart tube is a dynamic suction pump. Science 312, 751–753. 10.1126/science.1123775 16675702

[B27] Gjorevski N. Nelson C. M. (2010). The mechanics of development: Models and methods for tissue morphogenesis. Birth Defects Res. C Embryo Today. 90, 193–202. 10.1002/bdrc.20185 20860059 PMC3087175

[B28] Goenezen S. Rennie M. Y. Rugonyi S. (2012). Biomechanics of early cardiac development. Biomech. Model Mechanobiol. 11, 1187–1204. 10.1007/s10237-012-0414-7 22760547 PMC3475730

[B29] González-Rosa J. M. Guzmán-Martínez G. Marques I. J. Sánchez-Iranzo H. Jiménez-Borreguero L. J. Mercader N. (2014). Use of echocardiography reveals reestablishment of ventricular pumping efficiency and partial ventricular wall motion recovery upon ventricular cryoinjury in the zebrafish. PLoS ONE 9:e115604. 10.1371/journal.pone.0115604 25532015 PMC4274112

[B30] Granados-Riveron J. T. Brook J. D. (2012). The impact of mechanical forces in heart morphogenesis. Circ. Cardiovasc. Genet. 5, 132–142. 10.1161/CIRCGENETICS.111.961086 22337926

[B31] Gregg C. L. Butcher J. T. (2012). Quantitative *in vivo* imaging of embryonic development: opportunities and challenges. Differentiation 84, 149–162. 10.1016/j.diff.2012.05.003 22695188 PMC3694278

[B32] Hein S. J. Lehmann L. H. Kossack M. Juergensen L. Fuchs D. Katus H. A.. (2015). Advanced echocardiography in adult zebrafish reveals delayed recovery of heart function after myocardial cryoinjury. PLoS ONE 10:e0122665. 10.1371/journal.pone.0122665 25853735 PMC4390243

[B33] Hinton A. O. Jr. Yang Y. Quick A. P. Xu P. Reddy C. L. Yan X.. (2016). SRC-1 regulates blood pressure and aortic stiffness in female mice. PLoS ONE 11:e0168644. 10.1371/journal.pone.0168644 28006821 PMC5179266

[B34] Ho Y.-L. Shau Y.-W. Tsai H.-J. Lin L.-C. Huang J. Hsieh F.-J. (2002). Assessment of zebrafish cardiac performance using Doppler echocardiography and power angiography. Ultrasound Med. Biol. 28, 1137–1143. 10.1016/S0301-5629(02)00564-1 12401383

[B35] Hove J. R. Koster R. W. Forouhar A. S. Acevedo-Bolton G. Fraser S. E. Gharib M. (2003). Intracardiac fluid forces are an essential epigenetic factor for embryonic cardiogenesis. Nature 421, 172–177. 10.1038/nature01282 12520305

[B36] Howe K. Clark M. D. Torroja C. F. Torrance J. Berthelot C. Muffato M.. (2013). The zebrafish reference genome sequence and its relationship to the human genome. Nature 496, 498–503. 10.1038/nature12111 23594743 PMC3703927

[B37] Hu N. Christensen D. A. Agrawal A. K. Beaumont C. Clark E. B. Hawkins J. A. (2009). Dependence of aortic arch morphogenesis on intracardiac blood flow in the left atrial ligated chick embryo. Anat. Rec. (Hoboken). 292, 652–660. 10.1002/ar.20885 19322826

[B38] Huang Y. Wang X. Zhang J. Wu K. (2015). Impact of endocrine-disrupting chemicals on reproductive function in zebrafish (*Danio rerio*). Reprod. Domest. Anim. 50, 1–6. 10.1111/rda.12468 25529055

[B39] Instruments I. (2017). DFVS - Zebrafish Application Note - Noninvasive Cardiac Blood Flow Velocities. Available online at: http://indusinstruments.com/project/dfvs-zebrafish-application-note-v1/

[B40] James J. F. Hewett T. E. Robbins J. (1998). Cardiac physiology in transgenic mice. Circ. Res. 82, 407–415. 10.1161/01.RES.82.4.407 9506700

[B41] Jenkins M. W. Peterson L. Gu S. Gargesha M. Wilson D. L. Watanabe M.. (2010). Measuring hemodynamics in the developing heart tube with four-dimensional gated Doppler optical coherence tomography. J. Biomed. Opt. 15:066022. 10.1117/1.3509382 21198196 PMC3017576

[B42] Kang Y.-F. Li Y.-H. Fang Y.-W. Xu Y. Wei X.-M. Yin X.-B. (2015). Carbon quantum dots for zebrafish fluorescence imaging. Sci. Rep. 5:11835. 10.1038/srep11835 26135470 PMC4488761

[B43] Kari G. Rodeck U. Dicker A. (2007). Zebrafish: an emerging model system for human disease and drug discovery. Clin. Pharmacol. Ther. 82, 70–80. 10.1038/sj.clpt.6100223 17495877

[B44] Kendir C. van den Akker M. Vos R. Metsemakers J. (2018). Cardiovascular disease patients have increased risk for comorbidity: a cross-sectional study in the Netherlands. Eur. J. Gen. Pract. 24, 45–50. 10.1080/13814788.2017.1398318 29168400 PMC5795764

[B45] Kowalski W. J. Pekkan K. Tinney J. P. Keller B. B. (2014). Investigating developmental cardiovascular biomechanics and the origins of congenital heart defects. Front. Physiol. 5:408. 10.3389/fphys.2014.00408 25374544 PMC4204442

[B46] Lee J. Cao H. Kang B. J. Jen N. Yu F. Lee C. A.. (2014). Hemodynamics and ventricular function in a zebrafish model of injury and repair. Zebrafish 11, 447–454. 10.1089/zeb.2014.1016 25237983 PMC4172470

[B47] Lieschke G. J. Currie P. D. (2007). Animal models of human disease: zebrafish swim into view. Nat. Rev. Genet. 8, 353–367. 10.1038/nrg2091 17440532

[B48] Lindsey S. E. Butcher J. T. Yalcin H. C. (2014). Mechanical regulation of cardiac development. Front. Physiol. 5:318. 10.3389/fphys.2014.00318 25191277 PMC4140306

[B49] Liu C. C. Li L. Lam Y. W. Siu C. W. Cheng S. H. (2016). Improvement of surface ECG recording in adult zebrafish reveals that the value of this model exceeds our expectation. Sci. Rep. 6:25073. 10.1038/srep25073 27125643 PMC4850402

[B50] Locatelli P. Olea F. D. De Lorenzi A. Salmo F. Janavel G. L. V. Hnatiuk A. P.. (2011). Reference values for echocardiographic parameters and indexes of left ventricular function in healthy, young adult sheep used in translational research: comparison with standardized values in humans. Int. J. Clin. Exp. Med. 4, 258–264. 22140597 PMC3228581

[B51] Lucitti J. L. Tobita K. Keller B. B. (2005). Arterial hemodynamics and mechanical properties after circulatory intervention in the chick embryo. J. Exp. Biol. 208 (Pt 10), 1877–1885. 10.1242/jeb.01574 15879068

[B52] Mahler G. J. Frendl C. M. Cao Q. Butcher J. T. (2014). Effects of shear stress pattern and magnitude on mesenchymal transformation and invasion of aortic valve endothelial cells. Biotechnol. Bioeng. 111, 2326–2337. 10.1002/bit.25291 24898772 PMC4472486

[B53] Mammoto T. Ingber D. E. (2010). Mechanical control of tissue and organ development. Development 137, 1407–1420. 10.1242/dev.024166 20388652 PMC2853843

[B54] McLeish J. A. Chico T. J. Taylor H. B. Tucker C. Donaldson K. Brown S. B. (2010). Skin exposure to micro-and nano-particles can cause haemostasis in zebrafish larvae. Thromb. Haemost. 104, 797–807. 10.1160/TH09-06-0413 20174755

[B55] Midgett M. Rugonyi S. (2014). Congenital heart malformations induced by hemodynamic altering surgical interventions. Front. Physiol. 5:287. 10.3389/fphys.2014.00287 25136319 PMC4117980

[B56] Midgett M. Thornburg K. Rugonyi S. (2017). Blood flow patterns underlie developmental heart defects. Am. J. Physiol. Heart Circ. Physiol. 312, H632–H642. 10.1152/ajpheart.00641.2016 28062416 PMC5402020

[B57] Miura G. I. Yelon D. (2011). A guide to analysis of cardiac phenotypes in the zebrafish embryo. Methods Cell Biol. 101, 161–180. 10.1016/B978-0-12-387036-0.00007-4 21550443 PMC3292854

[B58] Packard R. R. S. Baek K. I. Beebe T. Jen N. Ding Y. (2017). Automated segmentation of light-sheet fluorescent imaging to characterize experimental doxorubicin-induced cardiac injury and repair. 7:8603. 10.1038/s41598-017-09152-x 28819303 PMC5561066

[B59] Pang K. L. Parnall M. Loughna S. (2017). Effect of altered haemodynamics on the developing mitral valve in chick embryonic heart. J. Mol. Cell. Cardiol. 108, 114–126. 10.1016/j.yjmcc.2017.05.012 28576718 PMC5529288

[B60] Parente V. Balasso S. Pompilio G. Verduci L. Colombo G. I. Milano G.. (2013). Hypoxia/reoxygenation cardiac injury and regeneration in zebrafish adult heart. PLoS ONE 8:e53748. 10.1371/journal.pone.0053748 23341992 PMC3547061

[B61] Parng C. Seng W. L. Semino C. McGrath P. (2002). Zebrafish: a preclinical model for drug screening. Assay Drug Dev. Technol. 1, 41–48. 10.1089/154065802761001293 15090155

[B62] Phoon C. K. Ji R. P. Aristizabal O. Worrad D. M. Zhou B. Baldwin H. S.. (2004). Embryonic heart failure in NFATc1-/- mice: novel mechanistic insights from *in utero* ultrasound biomicroscopy. Circ. Res. 95, 92–99. 10.1161/01.RES.0000133681.99617.28 15166096

[B63] Piliszek A. Kwon G. S. Hadjantonakis A. K. (2011). *Ex utero* culture and live imaging of mouse embryos. Methods Mol. Biol. 770, 243–257. 10.1007/978-1-61779-210-6_9 21805267 PMC3298811

[B64] Poon K. L. Brand T. (2013). The zebrafish model system in cardiovascular research: a tiny fish with mighty prospects. Glob. Cardiol. Sci. Pract. 2013, 9–28. 10.5339/gcsp.2013.4 24688998 PMC3963735

[B65] Roger V. L. Go A. S. Lloyd-Jones D. M. Adams R. J. Berry J. D. Brown T. M.. (2011). Heart disease and stroke statistics-2011 update: a report from the American heart association. Circulation 123, e18–e209. 10.1161/CIR.0b013e3182009701 21160056 PMC4418670

[B66] Samsa L. A. Yang B. Liu J. (2013). Embryonic cardiac chamber maturation: trabeculation, conduction, and cardiomyocyte proliferation. Am. J. Med. Genet. C Semin. Med. Genet. 163c, 157–168. 10.1002/ajmg.c.31366 23720419 PMC3723796

[B67] Spencer K. T. Kimura B. J. Korcarz C. E. Pellikka P. A. Rahko P. S. Siegel R. J. (2013). Focused cardiac ultrasound: recommendations from the American Society of Echocardiography. J. Am. Soc. Echocardiogr. 26, 567–581. 10.1016/j.echo.2013.04.001 23711341

[B68] Srivastava D. Olson E. N. (2000). A genetic blueprint for cardiac development. Nature 407, 221–226. 10.1038/35025190 11001064

[B69] Sun L. Lien C.-L. Xu X. Shung K. K. (2008). In vivo cardiac imaging of adult zebrafish using high frequency ultrasound (45-75 MHz). *Ultrasound Med. Biol* 34, 31–39. 10.1016/j.ultrasmedbio.2007.07.002 PMC2292109 17825980

[B70] Tobita K. Keller B. B. (2000). Right and left ventricular wall deformation patterns in normal and left heart hypoplasia chick embryos. Am. J. Physiol. Heart Circ. Physiol. 279, H959–H969. 10.1152/ajpheart.2000.279.3.H959 10993756

[B71] Tobita K. Schroder E. A. Tinney J. P. Garrison J. B. Keller B. B. (2002). Regional passive ventricular stress-strain relations during development of altered loads in chick embryo. Am. J. Physiol. Heart. Circ. Physiol. 282, H2386–H2396. 10.1152/ajpheart.00879.2001 12003850

[B72] Verkerk A. O. Remme C. A. (2012). Zebrafish: a novel research tool for cardiac (patho) electrophysiology and ion channel disorders. Front. Physiol. 3:255. 10.3389/fphys.2012.00255 22934012 PMC3429032

[B73] Wang L. W. Huttner I. G. Santiago C. F. Kesteven S. H. Yu Z. Y. Feneley M. P.. (2017). Standardized echocardiographic assessment of cardiac function in normal adult zebrafish and heart disease models. Dis. Model. Mech. 10, 63–76. 10.1242/dmm.026989 28067629 PMC5278526

[B74] Wang Y. Dur O. Patrick M. J. Tinney J. P. Tobita K. Keller B. B.. (2009). Aortic arch morphogenesis and flow modeling in the chick embryo. Ann. Biomed. Eng. 37, 1069–1081. 10.1007/s10439-009-9682-5 19337838

[B75] Watson L. E. Sheth M. Denyer R. F. Dostal D. E. (2004). Baseline echocardiographic values for adult male rats. J. Am. Soc. Echocardiogr. 17, 161–167. 10.1016/j.echo.2003.10.010 14752491

[B76] Wilson K. Baily J. Tucker C. Matrone G. Vass S. Moran C.. (2015). Early-life perturbations in glucocorticoid activity impacts on the structure, function and molecular composition of the adult zebrafish (*Danio rerio*) heart. Mol. Cell. Endocrinol. 414, 120–131. 10.1016/j.mce.2015.07.025 26219824 PMC4562295

[B77] Yalcin H. C. (2018). Hemodynamic studies for analyzing the teratogenic effects of drugs in the zebrafish embryo. Methods Mol. Biol. 1797, 487–495. 10.1007/978-1-4939-7883-0_27 29896711

[B78] Yalcin H. C. Amindari A. Butcher J. T. Althani A. Yacoub M. (2017). Heart function and hemodynamic analysis for zebrafish embryos. Dev. Dyn. 246, 868–880. 10.1002/dvdy.24497 28249360

[B79] Yalcin H. C. Shekhar A. McQuinn T. C. Butcher J. T. (2011). Hemodynamic patterning of the avian atrioventricular valve. Dev. Dyn. 240, 23–35. 10.1002/dvdy.22512 21181939 PMC3298748

[B80] Yalcin H. C. Shekhar A. Rane A. A. Butcher J. T. (2010). An *ex-ovo* chicken embryo culture system suitable for imaging and microsurgery applications. J. Vis. Exp. e2154. 10.3791/2154 21048670 PMC3185626

[B81] Yu Q. Leatherbury L. Tian X. Lo C. W. (2008). Cardiovascular assessment of fetal mice by *in utero* echocardiography. Ultrasound Med. Biol. 34, 741–752. 10.1016/j.ultrasmedbio.2007.11.001 18328616 PMC4275222

[B82] Zakaria Z. Z. Benslimane F. M. Nasrallah G. K. Shurbaji S. Younes N. N. Mraiche F.. (2018). Using zebrafish for investigating the molecular mechanisms of drug-induced cardiotoxicity. Biomed Res. Int. 2018:1642684. 10.1155/2018/1642684 30363733 PMC6180974

[B83] Zaragoza C. Gomez-Guerrero C. Martin-Ventura J. L. Blanco-Colio L. Lavin B. Mallavia B.. (2011). Animal models of cardiovascular diseases. J. Biomed. Biotechnol. 2011:497841. 10.1155/2011/497841 21403831 PMC3042667

