# Supplementary material for: Adaptation of a Mice Doppler Echocardiography Platform to Measure Cardiac Flow Velocities for Embryonic Chicken and Adult Zebrafish
Source: Front Bioeng Biotechnol. 2019 May 14;7:96. doi: 10.3389/fbioe.2019.00096 (PMC6527763; doi:10.3389/fbioe.2019.00096)
Supplement: Supplementary file 1 [file Data_Sheet_1.PDF]

Supplementary Material

1 Supplementary Figures

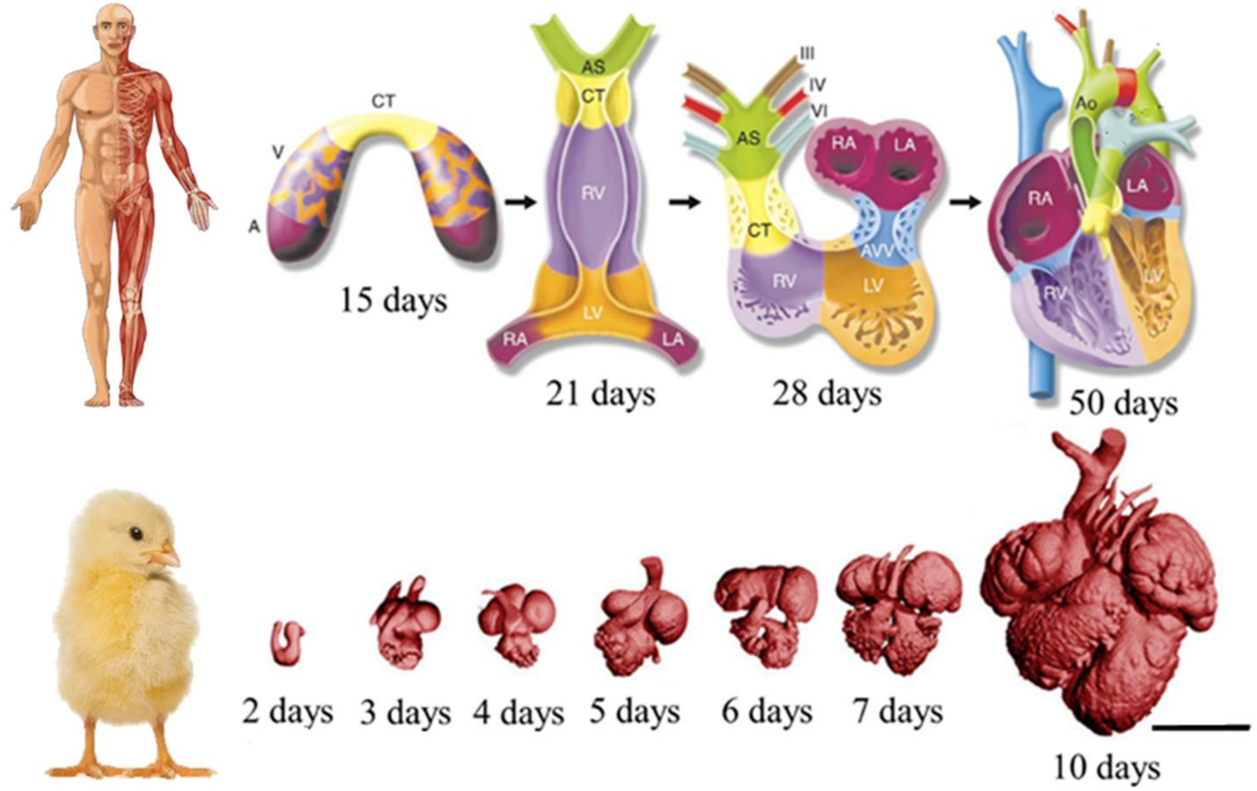

Supplementary Figure 1: Stages of heart development in human and chicken. Top panel: A schematic of cardiac morphogenesis in human. Lower panel: scaled micro-CT images of chicken embryonic hearts at representative days. Images were generated by our group in our previous work. Scale bar is 1 mm. A: atrium, V: Ventricle, CT: Conotruncal, AS: Aortic sac, RV: right vertical, LV: left vertical, RA: right atrium, LA: left atrium, Ao: Aorta. *Adopted from (Srivastava and Olson, 2000; Bharadwaj et al., 2012).*

Bharadwaj, K. N., Spitz, C., Shekhar, A., Yalcin, H. C. and Butcher, J. T. (2012) 'Computational fluid dynamics of developing avian outflow tract heart valves', *Ann Biomed Eng*, 40(10), pp. 2212-27.

Srivastava, D. and Olson, E. N. (2000) 'A genetic blueprint for cardiac development', *Nature*, 407(6801), pp. 221-6.

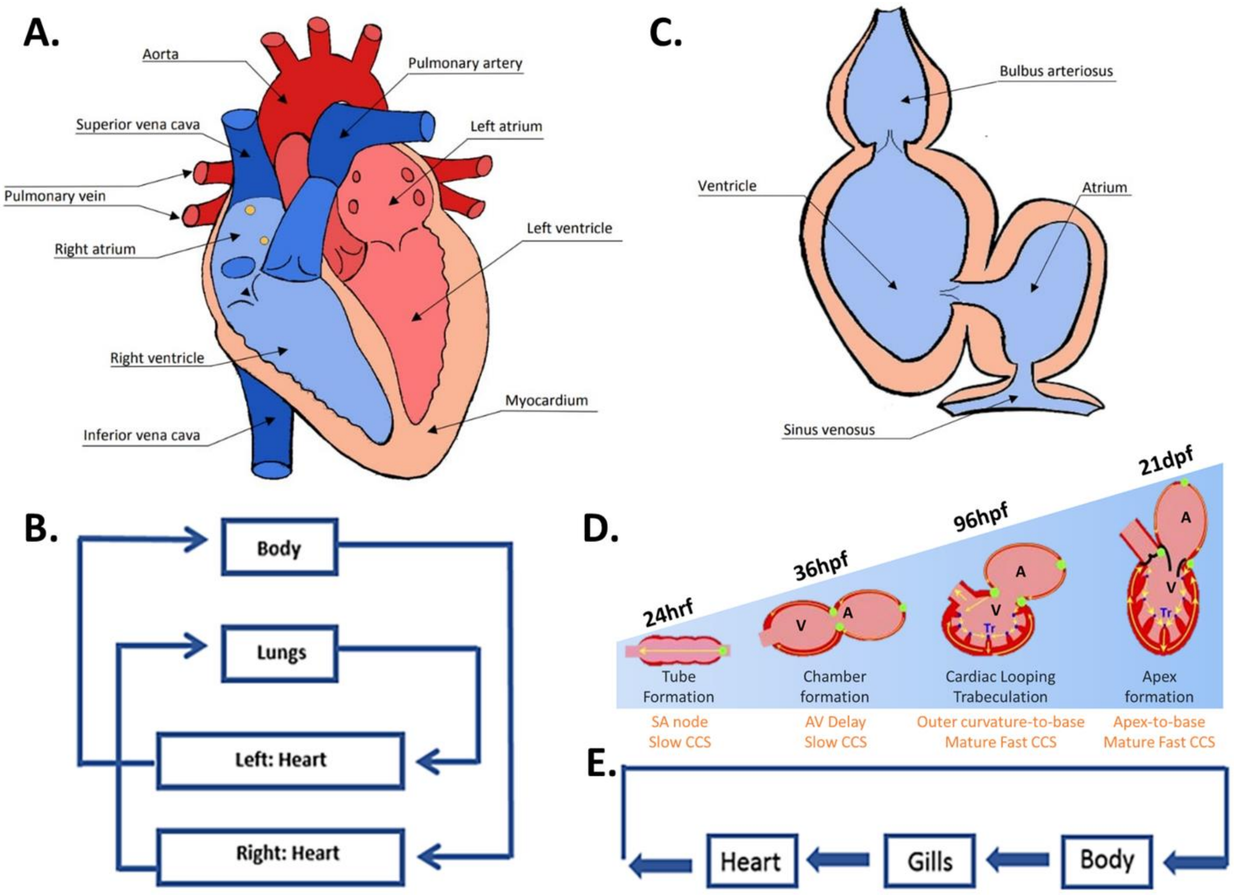

Supplementary Figure 2: The heart and blood circulation of humans and zebrafish. **A.** A four chambered human heart with a right atrium (RA), left atrium (LA), right ventricle (RV), left ventricle (LV) and **B.** the flow of blood through the body and chambers. **C.** A two chambered fish heart with a ventricle, atrium, sinus venosus and conus/bulbus arteriosus. **D.** Zebrafish heart starts as a tube which then loops and divides into a bulbus arteriosus, atrium and ventricle. Between the atrium and ventricle, valves form that prevent backflow of blood from the ventricle to the atrium. Initially, the contractions of the cardiac tube are peristaltic. Later, sequential contractions of the atrium and ventricle begin, which are initiated at the SA node located in the atrium. A conduction delay between the atrium and ventricle is observed around 48 hpf. A rudimentary apex-to-base conduction system appears at 96 hpf. The conduction system is almost completely developed by 5 days after fertilization (dpf), although some development of the fast conduction system continues as the apex of the heart matures until 21 dpf. Cardiac control through the autonomic nervous system also continues to develop after 96 hpf. **E.** In zebrafish, the blood flows direction from the body, through the sinus venosus, the atria, and ventricle, and from there to the bulbus arteriosus, through the gills for oxygenation, and back to the body.
